# The mountains of giants: an anthropometric survey of male youths in Bosnia and Herzegovina

**DOI:** 10.1098/rsos.161054

**Published:** 2017-04-12

**Authors:** Pavel Grasgruber, Stevo Popović, Dominik Bokuvka, Ivan Davidović, Sylva Hřebíčková, Pavlína Ingrová, Predrag Potpara, Stipan Prce, Nikola Stračárová

**Affiliations:** 1 Faculty of Sports Studies, Masaryk University, Kamenice 5, 62500 Brno, Czech Republic; 2 Faculty for Sport and Physical Education, University of Montenegro, Narodne omladine bb, 81400 Niksić, Montenegro; 3 Ekonomska škola, Ul. Vladimira Rolovica 2, Bar, Montenegro; 4 Department of Anthropology, Faculty of Science, Masaryk University, Kotlarska 2, 61137 Brno, Czech Republic; 5 Gimnazija Metković, Ul. kralja Zvonimira 10, 20350 Metković, Croatia

**Keywords:** Bosnia and Herzegovina, Dinaric Alps, height, genetics, nutrition

## Abstract

The aim of this anthropometric survey, conducted between 2015 and 2016 in Bosnia and Herzegovina (BiH), was to map local geographical differences in male stature and some other anthropometric characteristics (sitting height, arm span). In addition, to investigate the main environmental factors influencing physical growth, the documented values of height would be compared with available nutritional and socioeconomic statistics. Anthropometric data were collected in 3192 boys aged approximately 18.3 years (17–20 years), from 97 schools in 37 towns. When corrected for population size in the examined regions, the average height of young males in BiH is 181.2 cm (181.4 cm in the Bosniak-Croat Federation, 180.9 cm in Republika Srpska). The regional variation is considerable—from 179.7 cm in the region of Doboj to 184.5 cm in the region of Trebinje. These results fill a long-term gap in the anthropological research of the Western Balkans and confirm older reports that the population of the Dinaric Alps is distinguished by extraordinary physical stature. Together with the Dutch, Montenegrins and Dalmatians, men from Herzegovina (183.4 cm) can be regarded as the tallest in the world. Because both nutritional standards and socioeconomic conditions are still deeply suboptimal, the most likely explanation of this exceptional height lies in specific genetic factors associated with the spread of Y haplogroup I-M170. The genetic potential for height in this region could then be the greatest in the world. Future studies should further elucidate the roots of this intriguing phenomenon, which touches an important aspect of human biodiversity.

## 1. Introduction

The region of the Dinaric Alps has historically been renowned for the remarkable body size of its inhabitants [1,2] and from the anthropological view, it may well be the most fascinating area in the world. At the end of the 19th century, the mean height of people from Montenegro was 177 cm and those from Herzegovina 175–176 cm [1]. Bosnian soldiers in the Austro-Hungarian army in 1895 were 172.4 cm tall [3]. These were unusually high values in the period, when the majority of European countries barely reached 170 cm. Our previous study [4] pointed out the peculiar genetic background of this regional phenomenon. More concretely, male height in 43 countries of Europe and USA has a remarkable correlation with the frequency of Y haplogroup (paternal lineage) I-M170 (*r* = 0.65, *p* < 0.001), which is regarded as the genetic legacy of the Upper Paleolithic Gravettian culture. This Y haplogroup has two main frequency peaks among the Germanic-speaking nations of North-Central and Northern Europe, and in the Western Balkans [5].

The Western Balkan lineages consist mainly of a specific sub-branch I2a1-P37.2 and more concretely of I2a1b-M423. The available data indicate that the frequency of I-M170 is over 70% in Herzegovina, approximately 50% in central and eastern regions of BiH, approximately 35% in the north of BiH, 63% in Dubrovnik and 38% in Montenegro [6–10]. The global peak of I-M170 frequencies was documented among Croats in Herzegovina (73.3%; 71.1% I2a1-P37.2) [8]. The average frequency of I-M170 in BiH reported by the same authors is 53.1% (49.2% I2a1-P37.2).

The regional variation in height within the Western Balkans has historically been similarly large as in the case of I-M170. According to the data from the late nineteenth century [1], male stature decreased quite sharply from 175–176 cm in Herzegovina to 171–172 cm in northern Bosnia and 166–171 cm on the Adriatic coast. To our knowledge, no later anthropological research has dealt with the regional differences in the Dinaric Alps in detail. Especially in the late twentieth century, there was a long-term lack of any data. More information started to come in at the beginning of the twenty-first century. The study of Pineau *et al*. [11] reported a mean male height of 183.8 cm (*n* = 1253) in Dalmatia and 185.2 cm (*n* = 819) in Herzegovina. Later, the authors measured even Montenegro (183.1 cm, *n* = 908) (J.-C. Pineau 2013, personal communication). The work of Pineau *et al*. again drew deserved attention to the anthropology of the Dinaric Alps, but it did not report any detailed regional data on height, did not measure Bosnia, and furthermore, the measured boys were only 17-year-old high schoolers.

A more recent, but much smaller survey conducted in the Bosniak-Croat Federation of BiH in 2012 (A. Pilav 2015, personal communication) documented a mean height of 182.1 cm (*n* = 210) in 20- to 25-year old men, but sufficient data were collected only in six cantons, with large interregional differences: from unimpressive values in Muslim cantons of Una-Sana (180.9 cm), Tuzla (181.1 cm) and Zenica-Doboj (180.8 cm), through remarkably high values in Central Bosnia (182.1 cm) and up to very high numbers in the cantons of Herzegovina-Neretva (184.4 cm) and Sarajevo (184.6 cm). The latest anthropometric study in BiH was performed in 2014–2015 [3] and measured 415 men aged 20 to 24 years, who reached a mean of 182.8 cm. However, this study again covered only six cantons of the Federation and the Brčko District. The most actual measurements of 18- to 19-year-old high school boys in Montenegro (in 2013) agree with the data of Pineau *et al*. and found a mean height of 183.2 cm (*n* = 710), with large differences between taller northwestern and shorter southeastern regions (S. Popović 2014, personal communication).

Therefore, with regard to the fragmentary and incomplete information about the height trend on the territory of BiH, we decided to conduct a large-scale survey that would cover the whole area of this country and enable a detailed mapping of the regional differences in stature and some other anthropometric characteristics (sitting height, arm span). In addition, to investigate the main environmental factors influencing physical growth, the documented values of height would be compared with available nutritional and socioeconomic statistics. The primary hypothesis of our study was that the unusual height in the Dinaric Alps is mainly determined by genetics, and environment would play a secondary role. In any case, the identification of regions with the highest values of height would subsequently enable a better study of the Dinaric phenomenon.

## 2. Methods

The research in BiH was part of a larger anthropological project undertaken in collaboration with the University of Montenegro on the territory of BiH, Dalmatia and Montenegro during the years 2015–2016. At present, this project already covers Bosnia, Herzegovina, the entire Adriatic coast of Croatia, Montenegro and Kosovo, where over 6000 boys have been measured so far, in addition to several thousands of girls.

### 2.1. Regional division

**Figure 1.**
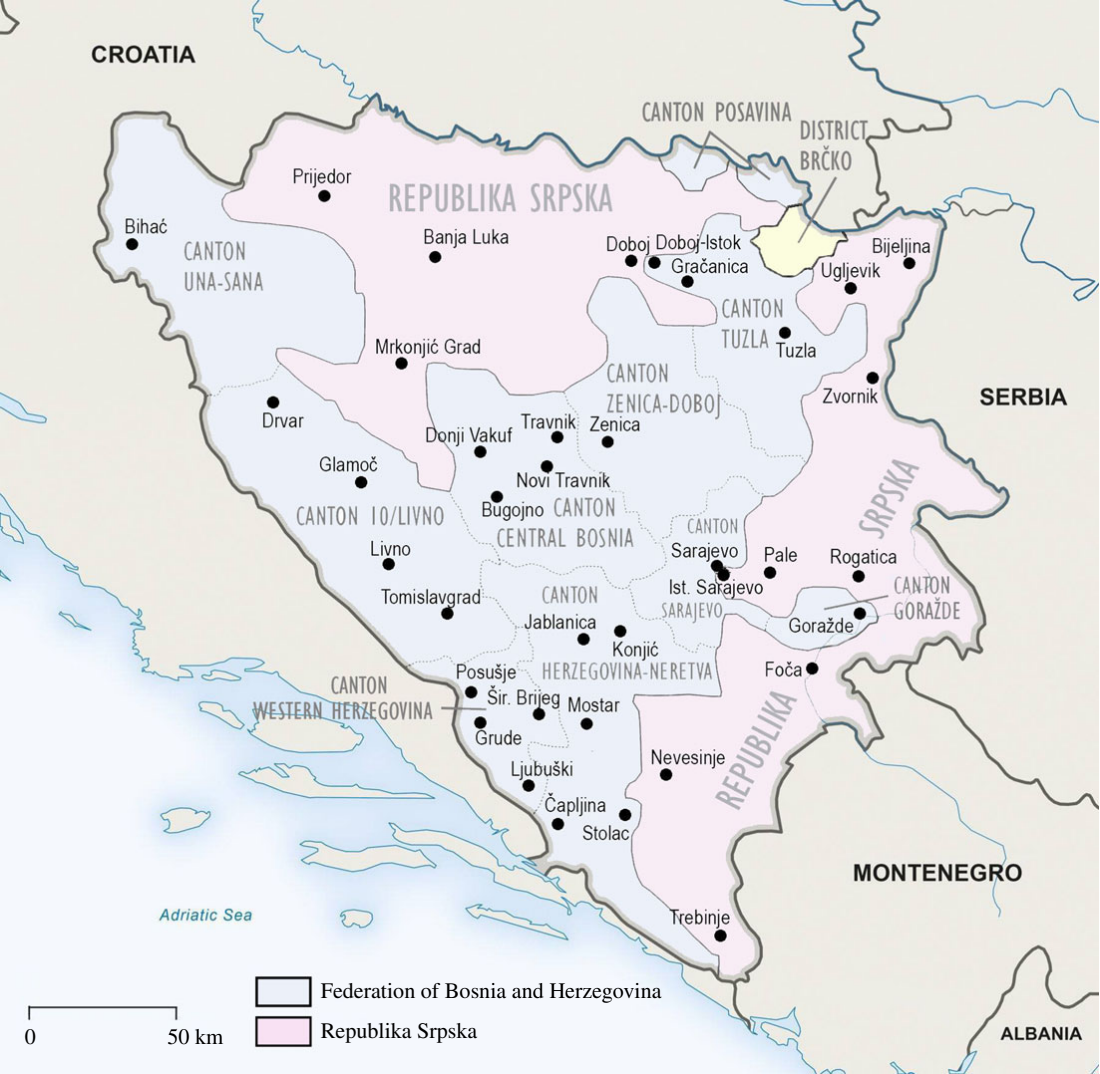
Political division of BiH and localities in which the measurements took place.

Before the start of the survey, in order to obtain maximally representative data covering the whole country, we divided the territory of BiH into regions of comparable size. This was easy in the case of the Bosniak-Croat Federation that consists of 10 cantons (see figure 1 and electronic supplementary material, figure S1): Una-Sana (Unsko-Sanski), Posavina (Posavski), Tuzla (Tuzlanski), Zenica-Doboj (Zeničko-dobojski), Goražde or Bosnian Podrinje (Bosansko-podrinjski kanton Goražde), Central Bosnia (Srednjobosanski), Herzegovina-Neretva (Hercegovačko-neretvanski), Western Herzegovina (Zapadno-Hercegovački), Sarajevo and Canton 10 (or Canton Livno).

The situation was more complicated in the case of Republika Srpska, which is divided into 64 municipalities and hence our division needed to be largely arbitrary. The western part of Republika Srpska was divided into three regions (Banja Luka, Doboj, Prijedor), based on the directory of high schools by the Pedagogical Institute of Republika Srpska (Adresar srednjih škola u Republici Srpskoj).^1^ Furthermore, we separated the region of Mrkonjić Grad from the region of Banja Luka due to its isolated position. The eastern part of Republika Srpska was divided into four regions: Bijeljina-Zvornik (including the towns of Bijeljina, Zvornik and Ugljevik), Romanija-Foča (including Pale, Rogatica and Foča), Istočno Sarajevo and Trebinje (including Trebinje and Nevesinje).

### 2.2. Target population

Due to the high time demands of this project tied with difficult terrain and the remoteness of some areas, and our goal to describe detailed regional differences in height within BiH, we concentrated on collecting sufficiently representative samples of one sex (males) and with few exceptions we did not include females. Because of the limitations of the previous surveys, we decided to target young men: high schoolers from the oldest grades, who were aged 18 years or older. However, because not all students from these grades were always available, the minimum age for the inclusion into our study was 17 years. Because our study included birth cohorts 1995–1998, the growth of the measured boys was only marginally influenced by the hardships of the Bosnian war, which officially ended in December 1995.

To ensure representativeness of our data, we always tried to measure the broadest spectrum of schools, from vocational to elite high schools (*gimnazija*). As a general rule, at least five schools in each big town were visited. In regions with small towns, the organization of the survey was more demanding, but we also tried to measure at least five different types of high schools. Our general goal was to incorporate sufficiently numerous samples of at least 200 individuals from each region and hence we always measured all boys who were available. Some contacted schools refused to participate due to organizational problems and in several other cases the directors did not receive consent from parents, but the responsiveness was generally very high (around 90%). Altogether, we visited 97 high schools in 37 towns (figure 1). Only the Brčko District and Canton Posavina were omitted, due to their relative insignificance and marginal geographical position. These two regions together make up only 3.7% of the total population.

### 2.3. Data collection

The measurements were conducted using two devices—a mobile stadiometer SECA 213 and a specially constructed apparatus designed for the measurement of height, sitting height and arm span. The mutual compatibility and accuracy of these devices was tested on a sample of 91 Czech high schoolers. The mean difference in the measured height was only 1.5 mm (180.10 versus 180.25 cm).

Before the measurements, the students completed a short questionnaire, which included an informed written consent, the date of birth, the place of residence and information regarding the university education of the students' parents. We supposed that there would be little difference between the place of birth and the current place of residence, because the students were mostly born after the end of the Bosnian war, when ethnic movements within BiH had already taken place.

The results obtained during the survey were subsequently analysed using the software Statistica 12. The data of height for each sample included means, standard deviations, medians, a range of extreme values, skewness, kurtosis and tests of normal distribution (Kolmogorov–Smirnov, Lilliefors and Shapiro–Wilk).

## 3. Results

Detailed results of the survey with the mean height of boys from all 97 high schools are included in the electronic supplementary material, table S1*a*,*b*. Regional averages, based on the self-reported place of residence, are presented in table 1. In summary, we measured 3207 boys aged 17–20 years in the territory of BiH, out of which 3192 resided in BiH and 15 boys came from other countries, mostly from the diaspora in Germany (*n* = 8) or from the neighbouring Serbia (*n* = 4). The averages of female samples from five schools (*n* = 69) are listed in the electronic supplementary material, table S2.

**Table 1.** Regional averages of male height (based on the self-reported place of residence), skewness, kurtosis and tests of normal distribution. *Abbreviations:* K-S test, Kolmogorov–Smirnov test; Sh-W test, Shapiro–Wilk test.

region/canton	*n*	average height (cm)	median	min.	max.	skewness	kurtosis	K-S test	Lilliefors test	Sh-W test
Region Trebinje	193	184.5 ± 7.7	183.8	163.1	206.6	0.21	0.17	*p* < 0.20	*p* < 0.01*	*p* = 0.12
Canton Western Herzeg.	215	184.0 ± 6.6	183.4	162.4	204.0	0.23	0.45	*p* > 0.20	*p* > 0.20	*p* = 0.25
Region Istočno Sarajevo	55	184.0 ± 5.6	184.1	171.4	198.0	−0.11	−0.07	*p* > 0.20	*p* > 0.20	*p* = 0.85
Canton 10/Livno	223	183.7 ± 6.7	183.9	162.9	200.8	−0.06	−0.05	*p* > 0.20	*p* > 0.20	*p* = 0.97
Canton Herzeg.-Neretva	352	182.8 ± 6.6	183.4	160.9	200.6	−0.15	0.41	*p* > 0.20	*p* < 0.10	*p* = 0.18
Canton Sarajevo	343	181.7 ± 6.7	182.0	159.7	205.3	−0.09	0.56	*p* > 0.20	*p* > 0.20	*p* = 0.41
Canton Central Bosnia	155	181.7 ± 6.5	182.3	167.0	203.3	−0.21	0.13	*p* > 0.20	*p* > 0.20	*p* = 0.46
Canton Zenica-Doboj	230	181.4 ± 5.9	181.2	167.6	200.3	0.15	0.07	*p* > 0.20	*p* > 0.20	*p* = 0.68
Region Bijeljina-Zvornik	229	181.2 ± 6.8	181.2	163.9	200.0	−0.12	0.13	*p* > 0.20	*p* > 0.20	*p* = 0.54
Region Romanija-Foča	98	181.0 ± 6.3	180.8	166.8	196.3	0.06	−0.30	*p* > 0.20	*p* < 0.20	*p* = 0.46
Region Prijedor	192	180.9 ± 7.2	180.1	164.9	201.0	0.53	0.14	*p* > 0.20	*p* < 0.10	*p* = 0.002*
Canton Goražde	65	180.6 ± 6.3	181.7	164.8	192.0	−0.60	−0.18	*p* > 0.20	*p* < 0.10	*p* = 0.050*
Region Banja Luka	170	180.5 ± 6.6	180.7	165.3	200.4	0.31	0.29	*p* > 0.20	*p* > 0.20	*p* = 0.16
Region Mrkonjić Grad	80	180.3 ± 6.2	180.2	167.2	197.2	0.15	−0.15	*p* > 0.20	*p* > 0.20	*p* = 0.83
Canton Una-Sana	193	180.0 ± 6.8	179.7	161.0	199.0	0.08	0.06	*p* > 0.20	*p* > 0.20	*p* = 0.85
Canton Tuzla	232	180.0 ± 5.8	180.0	165.3	194.9	−0.03	−0.05	*p* > 0.20	*p* > 0.20	*p* = 0.41
Region Doboj	167	179.7 ± 6.6	179.4	164.5	199.6	0.19	−0.08	*p* > 0.20	*p* > 0.20	*p* = 0.80
*Bosnia total*	*2209*	*180.9 ± 6.5*	*180.9*	*159.7*	*205.3*	*0.09*	*0.13*	*p > 0.20*	*p > 0.20*	*p = 0.20*
*Herzegovina total*	*983*	*183.6 ± 6.9*	*183.5*	*160.9*	*206.6*	*0.07*	*0.34*	*p > 0.20*	*p < 0.10*	p = 0.046*
*Federation total*	*2008*	*181.9 ± 6.6*	*182.0*	*159.7*	*205.3*	*0.02*	*0.24*	*p > 0.20*	*p > 0.20*	*p = 0.24*
*Republika Srpska total*	*1184*	*181.4 ± 7.0*	*181.1*	*163.1*	*206.6*	*0.25*	*0.16*	*p > 0.20*	p < 0.05*	p < 0.001*
*BiH total*	*3192*	*181.7 ± 6.8*	*181.6*	*159.7*	*206.6*	*0.11*	*0.20*	*p > 0.20*	*p < 0.10*	p = 0.006*

*Significantly different from the normal distribution of values.

### 3.1. Average height and regional variation in height

The average height of 3192 domestic boys was 181.7 ± 6.8 cm and their average age was 18.3 ± 0.3 years (17.0–20.6 years). However, regional differences are considerable and hence demographic differences are also important (electronic supplementary material, table S3*a*). If we take population size in each region into account, the average height drops to 181.2 cm (electronic supplementary material, table S3*b*), which still would be one of the very highest values in the world, being significantly surpassed only by the Netherlands (183.8 cm), Montenegro (183.2 cm) and Iceland (181.8 cm), and roughly on the same level as Sweden, Lithuania, Estonia, Serbia or the Czech republic (see our previous paper [4]). After this correction, the average height is 181.4 cm in the Federation and 180.9 cm in Republika Srpska.

**Figure 2.**
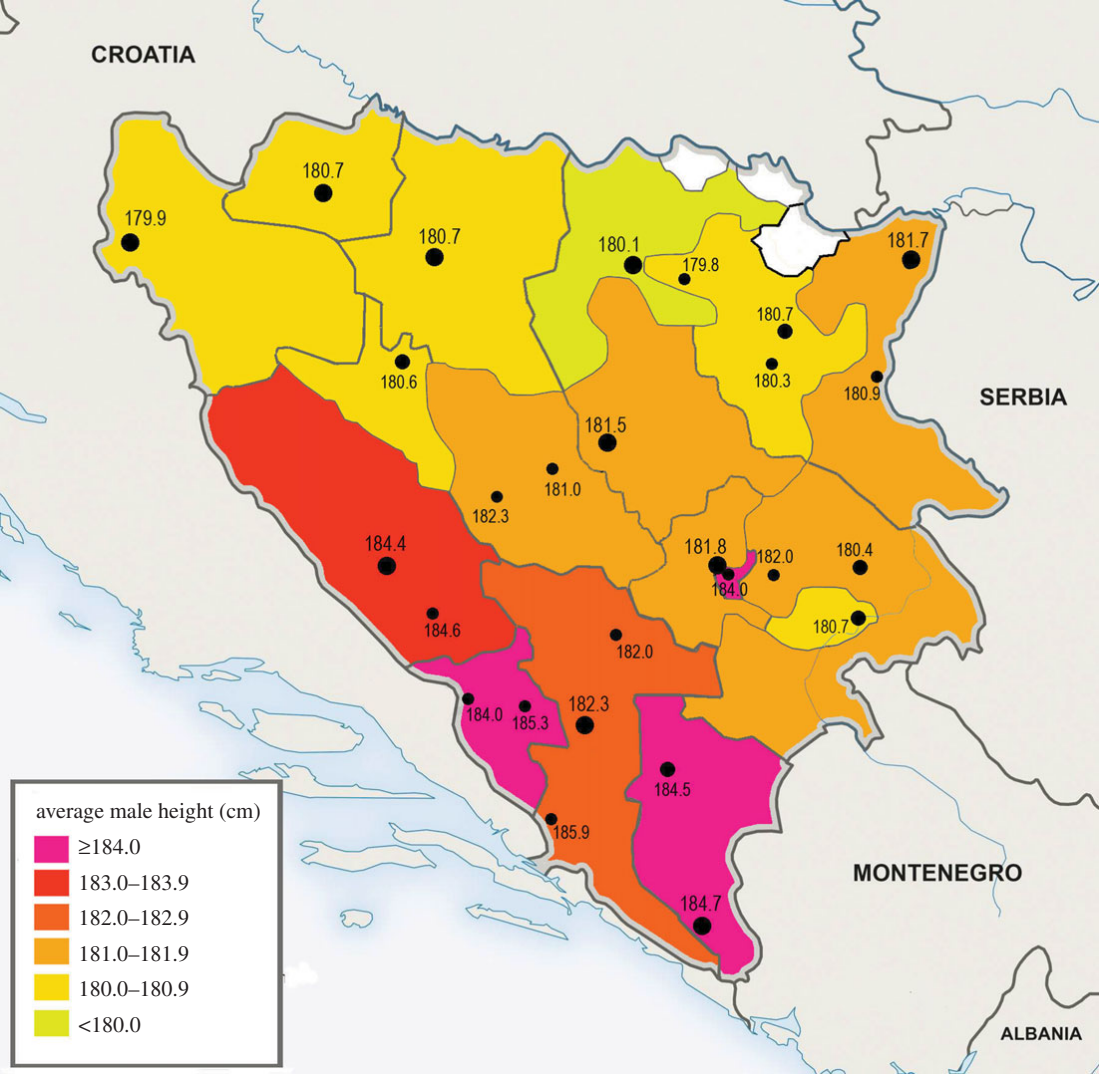
Regional averages of male height in Bosnia and Herzegovina (based on the self-reported place of residence), including average male height in individual 27 towns (table 2).

The lowest and highest averages in the 17 regions of BiH differ by 4.8 cm—from 179.7 in the region of Doboj to 184.5 cm in the region of Trebinje (table 1, figure 2). Apparently, the tallest statures approaching or exceeding 185 cm can be found in a narrow band stretching between Trebinje and Livno, and especially in the region of Trebinje and the area between Čapljina-Tomislavgrad (table 2). Nevertheless, the average height in the four Herzegovinian regions, including Canton 10/Livno, is only 183.6 cm and it further slightly decreases to 183.4, when corrected for population size. This is due to the lower mean documented in Canton Herzegovina-Neretva (182.8 cm) and particularly in Mostar (182.3 cm). The average height in Bosnia is considerably lower—180.9 cm (or 180.8 cm, when corrected for population size).

**Table 2.** Average male height in 27 towns (based on the self-reported place of residence). Only towns with at least 20 measured individuals were included.

region	town	*n*	Av. height (cm)
Canton Western Herzegovina	Čapljina	21	185.9
Canton Western Herzegovina	Široki Brijeg	38	185.4
Region Trebinje	Trebinje	120	184.7
Canton 10/Livno	Tomislavgrad	37	184.6
Region Trebinje	Nevesinje	71	184.5
Canton 10/Livno	Livno	101	184.4
Region Istočno Sarajevo	Istočno Sarajevo	55	184.0
Canton Western Herzegovina	Posušje	37	184.0
Canton Herzegovina-Neretva	Mostar	174	182.3
Canton Central Bosnia	Bugojno	49	182.3
Canton Herzegovina-Neretva	Konjić	44	182.0
Region Romanija-Foča	Pale	23	182.0
Canton Sarajevo	Sarajevo	324	181.8
Region Bijeljina-Zvornik	Bijeljina	122	181.7
Canton Zenica-Doboj	Zenica	157	181.5
Canton Central Bosnia	Novi Travnik	30	181.0
Region Bijeljina-Zvornik	Zvornik	28	180.9
Region Banja Luka	Banja Luka	147	180.7
Region Prijedor	Prijedor	110	180.7
Canton Tuzla	Tuzla	68	180.7
Canton Goražde	Goražde	57	180.7
Region Mrkonjić Grad	Mrkonjić Grad	69	180.6
Region Romanija-Foča	Rogatica	50	180.4
Canton Tuzla	Živinice	20	180.3
Region Doboj	Doboj	108	180.1
Canton Una-Sana	Bihać	173	179.9
Canton Tuzla	Gračanica	40	179.8

Tests of normal distribution show that almost all samples from 17 regions have a normal distribution of values (table 1). The Shapiro–Wilk test, which is regarded as the most sensitive [12], identifies only two exceptions at or below the significance level of *p* = 0.05. The first one is the small sample from Canton Goražde (*n* = 65), which is skewed to the left tail of the bell curve. The opposite case is the sample from the region of Prijedor (*n* = 192), which includes an unexpectedly large number of very tall individuals. These irregularities may potentially underestimate or overestimate the true regional mean, but the results are not fundamentally different from the neighbouring areas. The region of Trebinje, which reaches the tallest mean (184.5 cm), is also characterized by a large percentage of very tall boys, as indicated by a smaller median (183.8 cm) and the result of the Lilliefors test (*p* < 0.01). With regard to the large regional differences in height, it is not surprising that various combinations of regional samples produce abnormal shapes of the bell curve, especially in Republika Srpska.

It is remarkable that except for the geographically limited areas along the western border of Herzegovina, we could not collect any other data that would justify the high mean of 185.2 cm reported in Herzegovina by Pineau *et al*. [11]. At the same time, our sample from Herzegovina was even larger and the measured boys were on average 1 year older. Even without Canton 10/Livno, the mean of Herzegovina would not change—183.6 cm (*n* = 760). The reasons for this discrepancy are unclear. The possibility that height in Herzegovina would drop by approximately 1.5 cm during a single decade does not appear to be likely. As far as we know, not all the data listed by Pineau *et al*. were measured by the authors themselves and they were often obtained from teachers of physical education via personal communication. This suggests that they may have been influenced by non-standard conditions. Still, young men from Herzegovina (183.4 cm) are among the very tallest in the world and could be matched only by the Dutch (183.8 cm; [13]), Montenegrins (183.2 cm; S. Popović 2014, personal communication) and Dalmatians (approx. 183.5 cm; our preliminary unpublished results). The data that we have available show that very tall statures around 184 cm occur in a belt stretching across the territory of three West Balkan countries—from the western and northern municipalities of Montenegro through Herzegovina to the Croatian counties of Dubrovnik-Neretva, Split-Dalmatia and Šibenik-Knin.

In accordance with the data reported by Coon [1], the values of average height decrease in the direction to the north from Herzegovina, and the decrease is especially steep (183.7 to 180.3 cm) between the Croatian-speaking Canton 10/Livno and the region of Mrkonjić Grad in Republika Srpska, which are separated by the mountain ranges of Vitorog and Cincar (approx. 2000 m above sea level). The decrease of height between Mostar and cantons Sarajevo/Central Bosnia is more gradual (182.3 to 181.7 cm). Interestingly, our mean from the city of Sarajevo (181.8 cm, *n* = 324) differs markedly from the data provided by A. Pilav (184.6 cm, *n* = 47). Apparently, the problem must be sought in the much smaller sample size of the latter study. On the other hand, we did measure an unusually tall mean in the Serbian part of Sarajevo – Istočno Sarajevo (184.0 cm, *n* = 55). Although the sample was also relatively small, the boys came from two different schools and their tallness was striking. According to the information that we got from the Town Hall of Istočno Sarajevo, a large part of the residents in this town (as many as 70–80%) are historical migrants from Herzegovina and Western Montenegro.

The height in Canton Zenica-Doboj and in the regions of Romanija-Foča and Bijeljina-Zvornik is similar to Sarajevo and only slightly shorter, 181.0–181.4 cm. In Canton Goražde, it falls to 180.6 cm. Together with the region of Mrkonjić Grad and the northern part of Canton 10/Livno, the region of Goražde and Romanija-Foča is the most remote and the least populated in the whole country (electronic supplementary material, figures S2 and S3). The demographic and geographical conditions thus made our research particularly difficult. Actually, the sample from Romanija-Foča and Goražde includes only 163 boys altogether. However, despite the abnormal skewness of the small sample from Goražde, the averages from schools in Pale (181.4 cm), Rogatica (180.5 cm) and Goražde (180.5 cm) were quite similar, so we do not think that our results would be fundamentally distorted by the small sample size. Furthermore, the regions of Goražde and Romanija-Foča together make up only 3.5% of the total population of BiH, so their influence on the national average would be minimal. A potentially blank space remains in the region of Foča, where we found an average height of 183.1 cm, based on a mere 11 boys.

The shortest men in BiH can be found in the north and especially in the northwest, which again agrees with the information of Coon [1]. The region of Doboj was the only one with a mean below 180 cm (179.7 cm). Despite these low values, the available data indicate that the secular trend of height increase in northern Bosnia is very fast. According to Hadžihalilović *et al*. [14], the average height of 18-year old boys from high schools in Tuzla was 176.8 cm in 1980 (*n* = 192), 177.9 cm in 1996 (*n* = 155) and 178.8 cm in 2003 (*n* = 88). Our sample from Tuzla high schools reached 180.5 cm (*n* = 148), which suggests that since 1996, the pace of the secular trend has been approximately 1.4 cm/decade. Because the region of Tuzla is the most populous in BiH (13.1% of the total population), the pace of the local secular trend will be very important for the future development of the nationwide average.

In summary, the distribution of height in BiH—with the tallest values in Herzegovina (over 182 cm), the moderate values in central and eastern regions (approx. 181–182 cm), and the shortest values in the north (approx. 180–181 cm)—mirrors differences documented in the past and reflects quite well the geographical distribution of Y haplogroup I-M170.

### 3.2. Body proportions

The data on body proportions were collected in 1850 boys (1399 in Bosnia, 451 in Herzegovina), with the exception of three regions (Goražde, Herzegovina-Neretva, Romanija-Foča). The number of boys in which body proportions were examined ranged from 62 in Canton Tuzla to 193 in Canton Una-Sana. Relative sitting height in Herzegovina (52.11%) was significantly lower (*p* < 0.001) than in Bosnia (52.48%) (*p* < 0.001). The values of relative arm span in Bosnia (100.6%) and Herzegovina (100.3%) significantly differed as well (*p* = 0.002) (table 3*a–c*). During our current survey of Czech high schoolers aged 17–19 years (*n* = 463), we documented similar values of arm span (100.6%) and sitting height (52.30%). The difference in height between Bosnia and Herzegovina is 2.7 cm, but the difference in sitting height is only 1.1 cm, which shows that most of the difference in height can be attributed to the longer legs of Herzegovinian boys.

**Table 3.** Age-related differences in male height and body proportions.

	height		body proportions					
age (years)	*n*	Av. height (cm)	*n*	Av. height (cm)	sitting height (cm)	sitting height (% height)	arm span (cm)	arm span (% height)
(*a*) BiH								
17	841	182.0 ± 6.7	402	181.9 ± 7.0	95.1 ± 3.5	52.30 ± 1.35	183.2 ± 7.9	100.72 ± 2.19
18	2113	181.6 ± 6.7	1270	181.4 ± 6.9	95.0 ± 3.3	52.40 ± 1.38	182.4 ± 7.9	100.53 ± 2.26
19	226	181.7 ± 7.6	173	181.4 ± 7.3	95.2 ± 3.4	52.55 ± 1.55	182.1 ± 8.7	100.38 ± 2.34
20	12	181.9 ± 6.0	5	183.7 ± 4.2	95.2 ± 4.2	51.82 ± 2.01	180.7 ± 7.4	98.36 ± 0.79
17–20	3192	181.7 ± 6.7	1850	181.5 ± 6.9	95.1 ± 3.3	52.39 ± 1.40	182.5 ± 8.0	100.55 ± 2.25
(*b*) Bosnia								
17	552	181.4 ± 6.6	282	181.4 ± 6.8	94.8 ± 3.3	52.32 ± 1.33	182.9 ± 7.7	100.86 ± 2.24
18	1481	180.8 ± 6.5	979	180.6 ± 6.6	94.8 ± 3.3	52.52 ± 1.28	181.7 ± 7.6	100.63 ± 2.21
19	168	180.1 ± 7.1	134	180.1 ± 6.9	94.6 ± 3.2	52.53 ± 1.34	180.8 ± 8.3	100.37 ± 2.34
20	8	182.0 ± 6.6	4	183.2	96.3	52.57	180.0	98.22
17–20	2209	180.9 ± 6.5	1399	180.7 ± 6.7	94.8 ± 3.3	52.48 ± 1.30	181.9 ± 7.7	100.64 ± 2.23
(*c*) Herzegovina								
17	289	183.2 ± 6.7	120	183.2 ± 7.3	95.7 ± 3.7	52.27 ± 1.42	183.9 ± 8.2	100.40 ± 2.04
18	632	183.6 ± 7.0	291	184.2 ± 7.1	95.7 ± 3.2	51.99 ± 1.62	184.6 ± 8.4	100.18 ± 2.40
19	58	186.4 ± 7.0	39	185.5 ± 7.5	97.5 ± 3.1	52.62 ± 2.15	186.3 ± 8.7	100.43 ± 2.35
20	4	181.8	1	185.7	90.7	48.84	183.7	98.92
17–20	983	183.6 ± 6.9	451	184.1 ± 7.2	95.9 ± 3.4	52.11 ± 1.63	184.6 ± 8.3	100.26 ± 2.30

When the means of body proportions in BiH are divided according to age (table 3*a*), relative sitting height tends to increase from 52.30% in 17-year olds to 52.55% in 19-year olds. On the other hand, the trend of the relative arm span goes in the opposite direction, from 100.7% in 17-year olds to 100.4% in 19-year olds. These contrasting tendencies could reflect unfinished growth in some individuals, because at this age physical growth is almost entirely due to trunk growth [15]. This assumption seemingly contradicts the findings in table 3*a*, where we do not observe any trends towards taller statures in older boys, but the results are undoubtedly influenced by the large regional differences in height. For example, 17-year old boys from Herzegovina make up 34.4% of all boys aged 17 years, but only 29.5% of boys aged 18–20 years.

In any case, our findings in Herzegovina accord with the previous anthropological research in Montenegro that highlighted a peculiar combination of relatively longer legs (52%) and relatively shorter arm span (101%) in the Dinaric highlanders [1]. The values of relative sitting height negatively correlate significantly with height in the 17 predefined regions of BiH (*r* = −0.68, *p* = 0.008). The same relationship is apparent at the individual level (*r* = −0.46, *p* < 0.001). In other words, tallness is associated with progressively longer legs relative to stature. An opposite pattern emerges in the comparison of height and relative arm span. Here, the tallest regions tend to have the relatively shortest arm span (*r* = −0.57, *p* = 0.034), but this trend is very weak in individuals (*r* = −0.04, *p* = 0.09).

### 3.3. The role of socioeconomic factors

Information about the education of parents was collected for 3169 boys (2186 in Bosnia, 983 in Herzegovina) and, quite as expected, this factor significantly influenced height (table 4). Higher education may reflect both higher living standards and better parental care. The latter factor was clearly reflected by the fact that height depended mainly on the education of mothers. The university education of mothers added +1.4 cm on its own (182.7 cm), while university-educated fathers contributed only +0.7 cm (182.0 cm). However, height reached the highest values when both parents were university educated (+1.9 cm; 183.2 cm), and the difference was highly statistically significant (*p* < 0.001) when compared with sons of parents without university education (181.3 cm). Provided that the population of BiH could reach the current living standards of such university-educated families, the average of the whole country would rise to ca. 182.8 cm. The effect of education and social status also clearly manifested in taller statures of boys from elite schools (*gimnazija*). In Herzegovina, boys from *gimnazija* routinely reached 185–186 cm, and in the shortest northern regions they were mostly approximately 182–183 cm tall. These numbers should be taken as an indication of the minimum potential that these populations could reach under optimal living conditions.

**Table 4.** Relationship between male height and the university education of parents.

	measured males					
	BiH total		Bosnia		Herzegovina	
parents (university education)	*n*	Av. height (cm)	*n*	Av. height (cm)	*n*	Av. height (cm)
neither parent	2093	181.3 ± 6.8	1480	180.5 ± 6.5	613	183.4 ± 6.6
father	790	182.5 ± 6.9	530	181.9 ± 6.7	260	183.8 ± 7.2
father only	441	182.0 ± 6.8	297	181.4 ± 6.5	144	183.3 ± 7.2
mother	635	182.9 ± 6.9	409	182.2 ± 6.5	226	184.3 ± 7.4
mother only	286	182.7 ± 6.7	176	181.7 ± 5.9	110	184.2 ± 7.7
one parent	727	182.2 ± 6.8	473	181.5 ± 6.3	254	183.7 ± 7.4
both parents	349	183.2 ± 7.0	233	182.6 ± 6.8	116	184.4 ± 7.2

In contrast, we did not find any significant differences between boys from urban areas of BiH (towns and cities proper with more than 25 000 inhabitants), who had only a slightly shorter height (181.5 cm, *n* = 1558) than boys from rural areas (towns and villages with less than 25 000 inhabitants), who were 181.9 cm tall (*n* = 1634; *p* = 0.12). The taller stature of rural boys is unusual, because the urban environment should provide superior living conditions. As we will show below, this paradox is due to the shorter height of boys from Mostar in Herzegovina. Urban boys in Bosnia (181.1 cm, *n* = 1264) tend to be taller than rural boys (180.6 cm, *n* = 945), as expected, although this difference does not reach statistical significance either (*p* = 0.051).

Other socioeconomic statistics taken from the 2013 Census [16] or requested from the Agency for Statistics of BiH [17] are often geographically or time limited, and hence they cannot be used for a long-term analysis in all the examined regions. Out of them, only average net wages (in KM, convertible marks, for 2013) showed some positive relationship with height in our predefined 17 regions (*r* = 0.43, *p* = 0.08; electronic supplementary material, figure S4*a*). Without the outlier sample of Sarajevo, this tendency even reaches significance (*r* = 0.61, *p* = 0.013). Somewhat surprisingly, unemployment in 17 regions (for 2013) does not correlate with height at all (*r* = 0.28, *p* = 0.28; electronic supplementary material, figure S4*b*), despite the fact that the current rates of unemployment in BiH are one of the highest in the world.^2^ This finding could be explained by the high degree of economic self-sufficiency. Although the proportion of households engaged in private, market-oriented agricultural activities (for 2013) does not correlate with unemployment in 17 regions of BiH (*r* = 0.23, *p* = 0.37), this relationship tends to be positive in four regions of Herzegovina (*r* = 0.90, *p* = 0.10). Total private agricultural activities negatively correlate strongly with net wages in 17 regions (*r* = −0.86, *p* < 0.001; electronic supplementary material, figure S4*c*) and they approach significance as a predictor of height in four regions of Herzegovina (*r* = 0.94, *p* = 0.06; electronic supplementary material, table S4). This suggests that people in regions with low wages largely rely on private agricultural production and, at least in Herzegovina, unemployed families appear to benefit from these activities.

### 3.4. Nutrition

In our previous papers [4,18], we stressed the key importance of nutrition (protein consumption) for children's growth. Readers are advised to check the results of these ecological studies, which are based on the first large-scale comparison of nationwide averages of body height with various nutritional and socioeconomic statistics, and whose results sometimes question current dietary guidelines. In the meantime, other authors also came to similar conclusions [19,20], using the same ecological approach and statistics from the FAOSTAT database.

**Figure 3.**
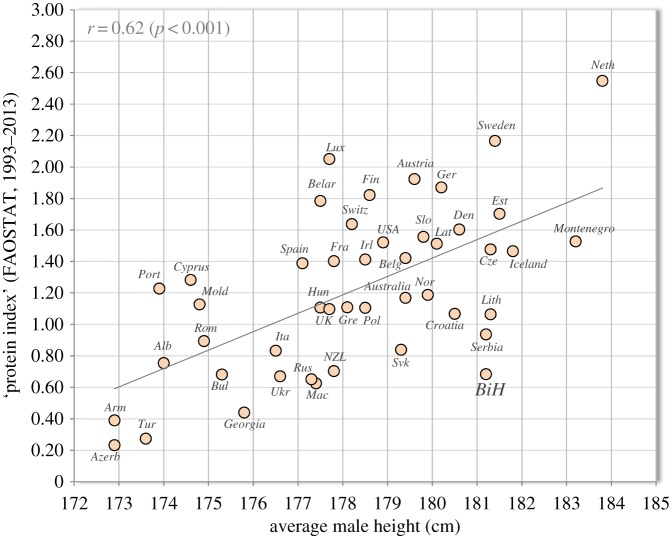
Relationship between male height and the ‘protein index’ (the ratio between high-quality proteins from dairy and pork, and low-quality proteins from wheat) in 46 countries of Europe and overseas (FAOSTAT, 1993–2013). *Note:* The graph contains updated values of height from our previous paper (see *Appendix: Methods* in Grasgruber *et al.* [18]) and recently updated/added data from Armenia (172.9 cm), Belgium (179.4 cm), Estonia (181.5 cm), Luxembourg (177.7 cm), Romania (174.9 cm), Serbia (181.2 cm) and Spain (177.1 cm). Protein consumption for Belgium and Luxembourg relates to the period 2000–2013, for Serbia and Montenegro to the period 2006–2013. The values of height from Australia, New Zealand and USA relate to the European (white) population.

A nutritional factor most strongly associated with height in Europe is the ‘protein index’—the ratio between proteins of the highest biological quality (such as those from dairy, pork, fish) and the lowest biological quality (cereals) [4]. In our present updated sample, the ratio between proteins from dairy and pork/wheat reaches the strongest relationship with male height in 46 countries of Europe and overseas (*r* = 0.62, *p* < 0.001; figure 3). This ratio is very low in BiH (0.68), much lower than in the world's tallest nation, the Netherlands (2.55). Furthermore, a regression model based on nutritional and socioeconomic statistics in 72 countries predicts only 175.9 cm for men in BiH (see [18]). Therefore, BiH is a striking outlier in almost all comparisons and nutritional and socioeconomic factors cannot explain the extraordinary values of body height in this country.

**Figure 4.**
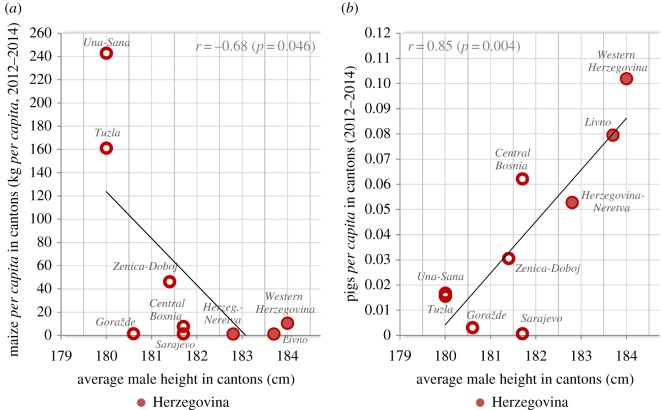
(*a*) Relationship between male height and maize production (kg/*per capita*/year) in nine cantons of the Federation. (*b*) Relationship between male height and pork production (pigs *per capita*) in nine cantons of the Federation.

Based on the mean food production in nine cantons for 2012–2014 (Kantoni u brojkama (Cantons in numbers), provided by the Federal Bureau of Statistics (Federalni zavod za statistiku), http://fzs.ba/ (S. Imamović 2016, personal communication)), adjusted for population size (the 2013 Census), we can say that male height in nine cantons of the Federation significantly correlates only with maize *per capita* (*p* = −0.68, *p* = 0.046; figure 4*a*), and pigs *per capita* (*r* = 0.85, *p* = 0.004; figure 4*b*). Although regional food production may not necessarily equal regional food consumption (which was not provided by any statistical office in BiH), these findings are noteworthy. The *per capita* consumption rates of maize in BiH are among the highest in Europe and maize has an extremely low protein quality, with a protein energy/total energy ratio that is even lower than that of wheat.^3^ The role of pigs *per capita* is even more intriguing, because the religious prohibition of pork in the diet of Bosniaks (Muslims) is the only fundamental difference that distinguishes the diets of Bosniaks and Croats. Indeed, pigs *per capita* correlate strongly negatively with the proportion of Bosniaks (*r* = −0.95, *p* < 0.001) and strongly positively with the proportion of Croats in nine cantons (*r* = 0.97, *p* < 0.001).

These data indicate that pork production is very closely associated with pork consumption at the regional level. Furthermore, the proportion of Croats also strongly predicts height in cantons (*r* = 0.93, *p* < 0.001), whereas height correlates strongly negatively with the proportion of Bosniaks (*r* = −0.94, *p* < 0.001) and non-significantly with the proportion of Serbs (*r* = 0.33, *p* = 0.39). Considering the key importance of pork in the human diet, which emerges from our ecological studies, these findings make sense and the absence of high-quality proteins from pork in the diet may contribute to shorter statures in predominantly Bosniak cantons.

The current trends of protein intake in BiH are positive, with increasing consumption rates of meat, dairy and total protein, and stagnating consumption of plant foods (electronic supplementary material, figure S5), but the speed of this improvement was clearly affected by the economic crisis in 2008.

### 3.5. Female height

As mentioned in the Methods section, information concerning females was much more limited, due to time constraints of the project and our goal to collect representative regional data at least from one sex. We performed measurements of female students only at five schools in three cantons of Herzegovina (electronic supplementary material, table S2). The average height of these 69 girls aged approximately 18 years was 169.4 ± 6.0 cm. None of these five samples was shorter than 168 cm—the averages ranged from 168.3 cm at Druga gimnazija Mostar to 171.0 cm at Gimnazija Tomislavgrad. The mean difference in height between boys and girls from the same school ranged from 9.5 to 14.9 cm (13.2 cm on average). Considering that the standard difference between male and female height in tall European nations is approximately 13 cm [21], the measured female values would fit the value of approximately 170 cm that could be estimated from the male data in Herzegovina.

## 4. Discussion

The present study is the largest anthropometric survey of the young male population of Bosnia and Herzegovina that has been conducted since 1895 (see [3]). It brings the first detailed information about the regional differences in height in the core area of the Dinaric Alps. One of the most important findings is the fact that the magnitude of these regional differences is considerable (4.8 cm) and it would find very few parallels in other countries of Europe. Therefore, regional data from BiH must always be taken with caution and cannot represent the whole country.

Although the final average for the entire country, corrected for population size in the examined regions (181.2 cm), is somewhat lower than we expected, it is still one of the highest values in the world. The means of central regions (particularly Canton Zenica-Doboj) were the closest to this value, and hence they could be used as a clue for the estimation of the secular trend in BiH in the future. Moreover, considering that our samples included 17-year-olds, it is possible that the height of fully mature men in BiH is even slightly higher. Overall, the average height in BiH has increased by 8–10 cm since the end of the nineteenth century (table 5). This increase may seem large, but it is relatively low when compared with highly industrialized nations of Western Europe (10–17 cm) [4]. These data can serve as another indication that the height of men in BiH is still far from their genetic maximum.

**Table 5.** Comparison between average male height listed by Coon (1939) and the present study.

	Coon (1939)^a^	present study	increase (cm)
Herzegovina	175–176	183.4	7.4–8.4
Sarajevo	174	181.8	7.8
Mostar	173	182.2	9.2
Travnik	173	181.7 (Central Bosnia)	8.7
Bihać	172	179.9	7.9
Banja Luka	172	180.7	8.7
Tuzla	171	180.7	9.7

^a^Data were taken from Capus (1895), Krauss (1885), Weisbach (1889; 1895).

Because neither nutrition nor socioeconomic factors can explain these extraordinary values of physical stature, the most likely explanation is genetic, which we already outlined in our previous paper [4]. Unfortunately, a discussion about the genetic background of the Dinaric phenomenon faces a lack of any research dealing with this problem. Perhaps the most interesting studies that recently addressed the genetics of height in Europe [22,23] did not include any samples from the Western Balkans. This unhappy omission may be ascribed to the long-term dearth of any anthropometric data from this region. Therefore, our present work can be very important for the direction of future research.

In the absence of any special studies dealing with autosomal DNA, we can assess this problem only indirectly, via regional frequencies of Y haplogroup I-M170, which are most concentrated in Herzegovina. Here, we also find the highest values of height. Herzegovina is separated from Bosnia by a chain of hardly penetrable, forested mountains (electronic supplementary material, figure S6), and this geographical barrier must have limited genetic flow from continental Europe. The age of the local dominant subclade I2a1b-M423 is estimated at approximately 5000–7500 years, which corresponds with the adoption of agriculture in the Balkans and the subsequent population growth [10]. Considering that I-M170 is associated with tall statures not only in the Balkans but also in Central and Northern Europe, the roots of this phenomenon are deeper and probably date back to the Upper Paleolithic (the Gravettian culture). The link between I-M170 and the Gravettian culture is based primarily on the fact that the Balkans served as a glacial refugium for Gravettian populations from Central Europe [24]. Indeed, the oldest sample of I-M170 documented in Europe belongs to a Gravettian male from Paglicci in Italy (34 580–31 120 calibrated years ago) [25]. Even more importantly, the unusually tall stature of Upper Paleolithic males from the Gravettian culture has been documented by archaeological findings. Dočkalova & Vančata [26] estimated a mean height of 176.3 cm in Gravettian males from Moravia (*n* = 15) and 182.7 cm in Gravettian males from the Central Mediterranean (*n* = 11). These are values that men from highly developed European countries reached only after the advance of the industrial revolution during the twentieth century.

**Figure 5.**
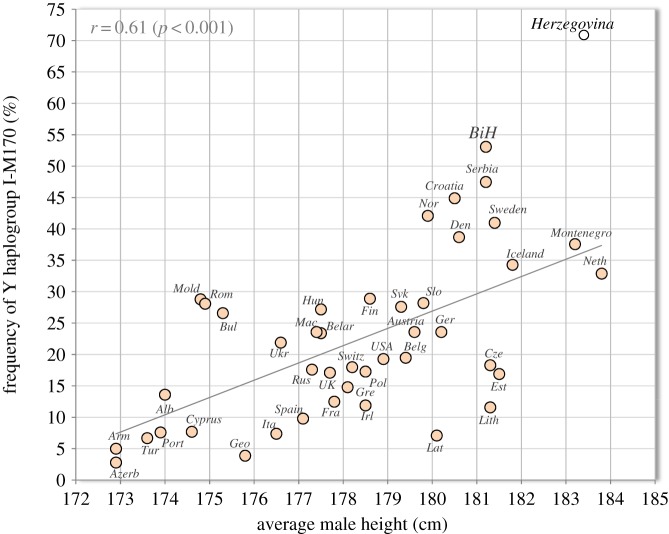
Relationship between average male height and the frequency of Y haplogroup I-M170 in 43 countries of Europe and USA. *Note:* The graph contains updated values of height (see figure 3). A separate regional sample of Herzegovina (70.9% I-M170; after Peričić *et al*. [7]) was added for an additional comparison.

Figure 5 shows that the frequency of Y haplogroup I-M170 in Herzegovina predicts a much higher value than that documented in the present study—over 185 cm and possibly close to 190 cm. These numbers would be by far the highest in the world, markedly surpassing even the well-nourished and wealthy Dutch, and as already mentioned, values over 185 cm can already be documented in some regions of southwestern Herzegovina and in students from elite high schools.

In addition to genetics, our results strongly suggest that the absence of high-quality proteins from pork in the diet of Bosniaks also contributes to geographical differences in height within BiH. This observation would support the key role of pork in the human diet, indicated by our previous studies, but its verification would require precise regional statistics of food consumption. Still, this nutritional factor can explain the notable anomaly of the ethnically divided Mostar in central Herzegovina, where we found considerably shorter values of height (182.3 cm) than in the nearby countryside and in other parts of Herzegovina. This is contrary to ‘common sense’, because the urban environment of the biggest town in Herzegovina should provide better access to high-quality food and healthcare. In accordance with our results at the regional level, we observed ethnic-related differences in height within this town: the mean of Croatian boys from Mostar (182.9 cm, *n* = 88), even without two elite schools, was taller than that of Bosniak boys (181.7 cm, *n* = 86), although the difference did not reach significance (*p* = 0.27). Recently, Kovačević *et al*. [27] demonstrated that historical admixture between Bosniaks and Turks during the Ottoman rule was very low, and hence an environmental explanation of these ethnic differences is more likely. Because Serbs and Montenegrins consume a lot of pork,^4^ it is possible that the same nutritional factors influence differences in height between (Bosniak) Sarajevo and (Serbian) Istočno Sarajevo.

Among the examined socioeconomic statistics, only average net wages are visibly associated with height at the regional level. This agrees with the importance of social status, evidenced by taller statures of boys from *gimnazija* and university-educated families. Other available socioeconomic factors do not play any obvious role, although it must be noted that the statistics were sometimes incomplete and did not allow a long-term analysis, which would cover the whole period of the boys' growth. For example, the important data on agricultural activities were available only from the 2013 Census. However, it is interesting that a regression model combining the current values of two socioeconomic (net wages, unemployment) and two nutritional (pigs *per capita* and maize *per capita*) variables can almost completely explain regional differences in height within the Federation (adj. *R*^2^ = 0.96, *p* < 0.001; table 6). This comparison highlights the key role of nutrition, but also the beneficial effect of net wages, which is complementary both to nutrition and private agricultural activities. Unemployment explains the smallest part of variance in most models and agricultural activities compromise the positive effect of pork production, possibly because they reflect low wages. Therefore, differences in height within the nine cantons do not necessarily require any genetic explanation. On the other hand, this finding cannot explain the height of Herzegovinians when compared with other countries, and considering that both pork consumption and net wages reach the highest values in Herzegovina, their role can be confounded by the presumed role of genetic predispositions.

**Table 6.** Regression models of average male height in nine cantons of the Federation. *Note:* The table displays *r*-values of partial correlations (i.e. correlations with height after controlling for all other independent variables included in the model), with asterisks indicating probability *p*-values.

independent variables	Model 1	Model 2	Model 3	Model 4	Model 5	Model 6	Model 7
pigs *per capita* (2012–14)		*r* = 0.91**	*r* = 0.87***	*r* = 0.94***	*r* = 0.95***	*r* = 0.98***	*r* = 0.98***
maize *per capita* (2012–14)			*r* = −0.73*			*r* = −0.83*	*r* = −0.83*
net wages (2013)	*r* = 0.83*				*r* = 0.84**	*r* = 0.90**	*r* = 0.93**
unemployment (2013)							*r* = −0.52
% households engaged in agricultural activities (total) (2013)	*r* = 0.81*			*r* = −0.75*			
% households engaged in agricultural activities (market-oriented) (2013)		*r* = −0.62					
strength of models (adj. R^2^)	0.598	0.774	0.828	0.839	0.895	0.961	0.964
(*p*-value)	(*p* = 0.03)	(*p* = 0.005)	(*p* = 0.002)	(*p* = 0.002)	(*p* < 0.001)	(*p* < 0.001)	(*p* < 0.001)

Significance level: *p < 0.05, **p < 0.01, ***p < 0.001.

Besides factors that we have already discussed, we should also mention the hypothesis of American anthropologist Carleton Coon, who speculated that in addition to genetics, the anomalous height of Dinaric highlanders is further elevated due to the above-average intake of minerals from the limestone bedrock of the local mountains [2]. The hypothesis linking tallness and physical robustness with high calcium intake is not unreasonable, because calcium supplementation during childhood leads to higher bone mass accumulation and faster bone growth [28]. Because testing the elevated content of calcium in the locally produced food sources would require laboratory tests, which we have not performed, Coon's hypothesis remains an interesting challenge for future research in the Dinaric area.

In summary, this survey in BiH may constitute a starting point for a more detailed investigation of the Dinaric phenomenon, which has so far been rather neglected in scientific studies. Because the genetic explanation appears to be most likely, such research would include the identification of specific alleles contributing to the exceptional body size of the local inhabitants. This would also clarify the mutual interaction between genetic and environmental factors in BiH, which we could not adequately address in the present study due to the lack of any autosomal DNA data related to height and the limitations of the available statistics.

## Supplementary Material

SUPPORTING INFORMATION-RSOS.pdf

## Supplementary Material

Supplementary dataset.xlsx

## Footnotes

1 Adresar srednjih škola u Republici Srpskoj. Available online at: http://www.rpz-rs.org

2 Accoding to the World Bank, the unemployment rate in BiH (for 2013) reached 27.4%, which was the third highest value in the world after Mauritania (31.1%) and Macedonia (29.0%).

3 According to the USDA.gov database, assuming 17.2 kJ per gram of protein, protein makes up 9.6% of total energy in white corn flour and 11.7% energy in white all-purpose wheat flour. The data in the FAOSTAT database indicate similar numbers: 9.5% for maize, 12.1% for wheat and 7.8% for rice (which was the main correlate of undernutrition in our previous study; Grasgruber *et al.* [18]). At the same time, the amino acid score of maize (0.46) is slightly lower than that of white wheat flour (0.48).

4 According to the FAOSTAT database (http://www.fao.org/faostat/en/#data/CL), the mean consumption of pork protein in Serbia & Montenegro between 1995 and 2005 was 15.7 g day^−1^, which was the third highest value in Europe after Austria and Spain. During the same period, the mean consumption of pork protein in Croatia (5.1 g day^−1^) and Bosnia & Herzegovina (1.5 g day^−1^) was very low.

## Data accessibility

Raw data from the survey are included in the electronic supplementary material.
