# Correction to ‘The mountains of giants: an anthropometric survey of male youths in Bosnia and Herzegovina’

**DOI:** 10.1098/rsos.170445

**Published:** 2017-05-31

**Authors:** Pavel Grasgruber, Stevo Popović, Dominik Bokuvka, Ivan Davidović, Sylva Hřebíčková, Pavlína Ingrová, Predrag Potpara, Stipe Prce, Nikola Stračárová

*R. Soc. open sci.* **4**, 161054. (Published 12 April 2017). (doi:10.1098/rsos.161054)

In figure 2, the height in Široki Brijeg is listed as 185.3 cm. The correct value is 185.4 cm. The corrected figure is shown below.


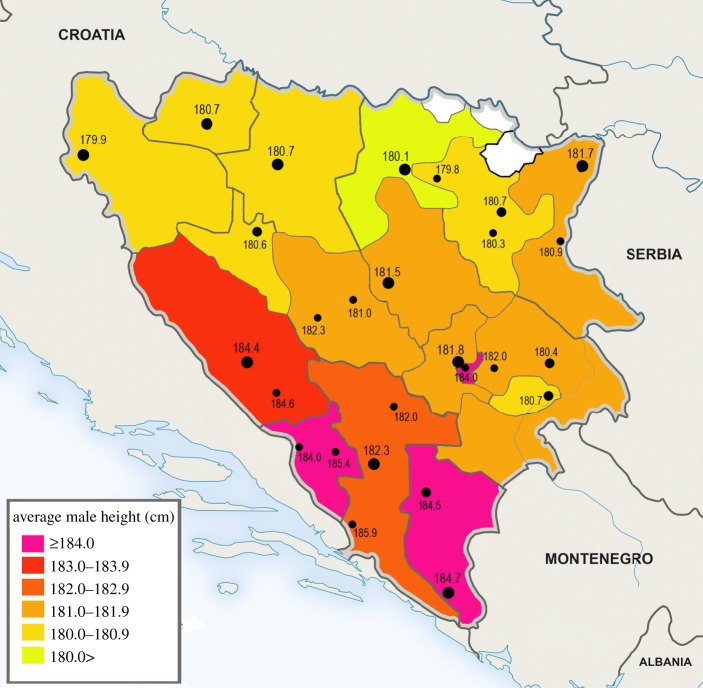


In table 2, the region of the town of Čapljina is mistakenly listed as ‘Canton Western Herzegovina’. The correct location is ‘Canton Herzegovina-Neretva’. The corrected table is shown below.

**Table 2.** Average male height in 27 towns (based on the self-reported place of residence). Only towns with at least 20 measured individuals were included.

region	town	*n*	av. height (cm)
Canton Herzegovina-Neretva	Čapljina	21	185.9
Canton Western Herzegovina	Široki Brijeg	38	185.4
Region Trebinje	Trebinje	120	184.7
Canton 10/Livno	Tomislavgrad	37	184.6
Region Trebinje	Nevesinje	71	184.5
Canton 10/Livno	Livno	101	184.4
Region Istočno Sarajevo	Istočno Sarajevo	55	184.0
Canton Western Herzegovina	Posušje	37	184.0
Canton Herzegovina-Neretva	Mostar	174	182.3
Canton Central Bosnia	Bugojno	49	182.3
Canton Herzegovina-Neretva	Konjić	44	182.0
Region Romanija-Foča	Pale	23	182.0
Canton Sarajevo	Sarajevo	324	181.8
Region Bijeljina-Zvornik	Bijeljina	122	181.7
Canton Zenica-Doboj	Zenica	157	181.5
Canton Central Bosnia	Novi Travnik	30	181.0
Region Bijeljina-Zvornik	Zvornik	28	180.9
Region Banja Luka	Banja Luka	147	180.7
Region Prijedor	Prijedor	110	180.7
Canton Tuzla	Tuzla	68	180.7
Canton Goražde	Goražde	57	180.7
Region Mrkonjić Grad	Mrkonjić Grad	69	180.6
Region Romanija-Foča	Rogatica	50	180.4
Canton Tuzla	Živinice	20	180.3
Region Doboj	Doboj	108	180.1
Canton Una-Sana	Bihać	173	179.9
Canton Tuzla	Gračanica	40	179.8

