# Supplementary material for: The mountains of giants: an anthropometric survey of male youths in Bosnia and Herzegovina
Source: R Soc Open Sci. 2017 Apr 12;4(4):161054. doi: 10.1098/rsos.161054 (PMC5414258; doi:10.1098/rsos.161054)
Supplement: SUPPORTING INFORMATION-RSOS.pdf [file rsos161054supp1.pdf]

Supplementary material

Table S1a. Averages of boys in the measured schools (Federation).

Region<br>TOWN, school	*n*	Height (cm)<br>Average (SD)	Range	Age (years)<br>Average (SD)	Range
**Canton Una-Sana**	***193***	**180.0 ±6.8**		**18.4 ±0.4**	
BIHAĆ, Gimnazija	*67*	181.9 ±6.6	167.7-199.0	18.3 ±0.5	17.5-19.2
BIHAĆ, Unsko-sanski koledž	*10*	179.8 ±5.8	170.9-190.6	18.4 ±0.4	17.9-19.1
BIHAĆ, Medicinska	*24*	179.5 ±6.6	161.0-191.3	18.5 ±0.3	17.7-19.3
BIHAĆ, Mješovita srednja	*87*	178.9 ±6.8	162.5-196.3	18.4 ±0.3	17.4-19.5
BIHAĆ, Katolički školski centar	*5*	177.6	171.7-185.9	18.2	17.8-18.8
**Canton 10/Livno**	***222***	**183.7 ±6.7**		**18.3 ±0.6**	
LIVNO, Ekonomska	*28*	185.5 ±6.0	174.9-198.0	18.8 ±0.3	18.1-19.7
LIVNO, Gimnazija	*44*	184.7 ±6.4	171.9-198.4	18.3 ±0.4	17.4-19.1
TOMISLAVGRAD, Gimnazija M. Marulića	*29*	184.3 ±7.3	166.7-196.0	18.0 ±0.5	17.0-18.7
DRVAR, Srednja škola	*11*	184.1 ±6.5	173.4-197.2	18.3 ±0.6	17.7-20.1
TOMISLAVGRAD, Strukovna	*22*	183.6 ±6.6	162.9-194.6	17.9 ±0.6	17.0-18.6
LIVNO, Strukovna S. Š. Kranjčevića	*71*	183.3 ±6.5	170.2-200.8	18.6 ±0.4	17.7-20.3
GLAMOČ, Srednja škola Tin Ujević	*17*	178.8 ±6.3	167.0-191.4	17.6 ±0.5	17.0-18.4
**Canton Western Herzegovina**	***215***	**184.0 ±6.6**		**18.2 ±0.5**	
ŠIROKI BRIJEG, Gimnazija	*28*	186.5 ±5.5	177.8-202.0	18.4 ±0.3	17.2-18.9
LJUBUŠKI, Gimnazija	*37*	185.2 ±6.9	172.2-204.0	18.4 ± 0.5	17.3-19.1
ŠIROKI BRIJEG, Strukovna	*47*	184.4 ±6.6	170.5-201.8	18.3 ±0.4	17.6-19.0
POSUŠJE, Strukovna	*29*	183.9 ±5.9	175.3-201.5	18.1 ±0.3	17.4-18.5
GRUDE, Srednja škola A. B. Šimića	*21*	183.1 ±7.1	173.1-197.4	17.8 ±0.3	17.1-18.4
POSUŠJE, Gimnazija	*27*	182.2 ±4.3	173.8-194.0	18.1 ±0.4	17.4-19.2
LJUBUŠKI, SSŠ Rudera Boskovića	*26*	181.5 ±8.3	162.4-194.7	18.3 ± 0.6	17.4-19.3
**Canton Herzegovina-Neretva**	***354***	**182.7 ±6.5**		**18.1 ±0.4**	
MOSTAR, Gradevinska (Croatian)	*17*	187.5 ±4.7	178.9-200.4	18.0 ±0.3	17.6-18.6
ČAPLJINA, Srednja škola	*22*	184.8 ±5.5	175.8-199.5	18.2 ±0.3	17.6-18.8
JABLANICA, Srednja škola	*26*	184.7 ±5.9	171.5-198.7	18.2 ±0.4	17.1-19.3
STOLAC, Srednja škola	*38*	183.5 ±6.4	178.3-195.6	18.2 ±0.4	17.6-18.5
MOSTAR, Turističko-ugostiteljska (Croatian)	*11*	182.6 ±7.6	164.9-196.2	18.1 ±0.4	17.5-18.7
MOSTAR, Elektrotehnička (Bosniak)	*57*	182.5 ±7.4	167.6-200.6	18.1 ±0.3	17.3-18.7
MOSTAR, Druga gimnazija (Bosniak)	*20*	181.9 ±4.7	173.0-192.5	18.1 ±0.3	17.6-18.5
MOSTAR, Ekonomska (Croatian)	*24*	181.8±6.0	170.7-193.9	18.4 ±0.5	17.3-19.0
KONJIČ, Srednja škola	*49*	181.7 ±6.9	160.9-195.0	18.2 ±0.5	17.1-19.8
MOSTAR, Prometna (Croatian)	*35*	181.5 ±6.3	169.1-194.9	18.2 ±0.4	17.5-19.7
MOSTAR, Strojarska (Croatian)	*30*	181.3 ±4.8	168.2-187.4	18.1 ±0.4	17.6-19.6
MOSTAR, Medicinska (Bosniak)	*25*	180.9 ±7.3	162.5-197.0	17.9 ±0.4	17.2-18.7
**Canton Central Bosnia**	***145***	**181.8 ±6.5**		**18.6 ±0.4**	
BUGOJNO, Tehnička	*20*	183.7 ±5.9	172.2-194.3	18.7 ±0.4	18.0-19.8
DONJI VAKUF, Mješovita srednja	*20*	182.0 ±6.1	171.4-192.7	18.2 ±0.5	17.3-19.4
NOVI TRAVNIK, Opća gimnazija	*54*	182.0 ±7.5	168.0-203.3	18.6 ±0.4	17.7-19.8
BUGOJNO, Gimnazija	*30*	181.2 ±5.9	170.9-192.5	18.5 ±0.3	17.8-19.1
TRAVNIK, Mješovita srednja	*21*	180.1 ±5.5	167.0-189.5	18.7 ±0.3	18.1-19.2

**Region**	*n*	Height (cm)		Age (years)	
TOWN, school		Average (SD)	Range	Average (SD)	Range
**Canton Zenica-Doboj**	***230***	**181.3 ±5.9**		**18.2 ±0.4**	
ZENICA, Opća gimnazija	*33*	183.5 ±5.6	174.5-196.4	18.0 ±0.5	17.0-18.7
ZENICA, Ekonomska	*16*	182.2 ±6.2	171.3-195.3	18.2 ±0.4	17.9-19.3
ZENICA, Muzička	*7*	181.1 ±5.9	172.0-188.2	18.1 ±0.4	17.3-18.7
ZENICA, Prva gimnazija	*45*	181.0 ±5.7	168.3-193.4	18.2 ±0.3	17.4-19.1
ZENICA, Tehnička	*116*	180.8 ±5.6	167.6-196.6	18.3 ±0.3	17.6-19.1
ZENICA, Druga gimnazija	*13*	180.7 ±7.3	169.2-200.3	18.0 ±0.4	17.5-19.1
**Canton Sarajevo**	***360***	**181.8 ±6.8**		**18.4 ±0.6**	
SARAJEVO, Druga gimnazija	*24*	184.0 ±5.4	172.6-194.0	18.1 ±0.3	17.4-18.7
SARAJEVO, Treća gimnazija	*28*	182.9±5.8	169.4-193.0	17.5±0.2	17.0-17.9
SARAJEVO, Gradevinsko-geodetska	*30*	182.5 ±5.0	169.8-196.7	18.7 ±0.6	17.6-20.0
SARAJEVO, Elektrotehnička za energetiku	*66*	182.3 ±6.9	159.7-198.1	18.6 ±0.4	17.7-19.7
SARAJEVO, Dizajnerska	*15*	182.3 ±5.0	173.9-192.2	18.7 ±0.5	18.0-20.2
SARAJEVO, Prominjenjih umjetnosti	*14*	182.1±6.6	175.2-202.0	18.7±0.4	18.3-19.6
SARAJEVO, Gimnazija Obala	*8*	181.8 ±5.1	172.9-187.1	18.4 ±0.7	17.6-19.8
SARAJEVO, Prva gimnazija	*22*	181.8±7.1	168.6-196.0	18.4±0.4	17.8-19.4
SARAJEVO, Ugostiteljsko-turistička	*50*	181.6±7.9	165.3-205.3	18.6±0.6	17.6-20.1
SARAJEVO, Katolički školski centar	*25*	180.7 ±7.5	167.5-199.3	18.3 ±0.4	17.6-19.7
SARAJEVO, Ekonomska	*26*	180.6 ±6.1	165.6-193.6	18.4±0.4	17.4-19.0
SARAJEVO, Metalskih zanimanja	*52*	180.3 ±7.0	159.9-194.4	18.3 ±0.6	17.3-19.9
**Canton Tuzla**	***233***	**179.9 ±5.9**		**18.1 ±0.4**	
TUZLA, Turističko-ugostiteljska	*3*	181.8	173.2-186.9	17.9	17.6-18.2
TUZLA, Ekonomska	*9*	181.7 ±1.9	179.0-185.0	17.9 ±0.5	17.0-18.6
GRAČANICA, Gimnazija	*21*	181.6 ±5.0	170.5-190.8	18.1 ±0.3	17.3-18.5
TUZLA, Medicinska	*43*	181.0 ±5.4	170.7-194.9	18.1 ±0.4	17.4-19.5
TUZLA, Rudarska	*41*	180.8 ±6.1	165.5-193.0	18.2 ±0.6	17.1-20.6
TUZLA, Gradevinsko-geodetska	*36*	179.6 ±6.0	168.6-194.0	18.1 ±0.4	17.2-18.7
TUZLA, Gimnazija Ismet Mujezinović	*17*	179.6 ±4.7	170.1-184.1	18.4 ±0.3	18.0-19.0
DOBOJ-ISTOK, Mješovita srednja	*20*	178.4 ±6.9	167.1-193.7	17.6 ±0.4	17.1-18.3
GRAČANICA, Mješovita srednja	*43*	177.8 ±6.1	165.3-191.1	18.3 ±0.2	18.0-18.7
**Canton Goražde (Bosnian-Podrinje)**	***69***	**180.5 ±6.2**		**18.1 ±0.4**	
GORAŽDE, Tehnička Hasib Hadžović	*49*	181.5 ±5.6	168.3-192.0	18.0 ±0.4	17.7-19.4
GORAŽDE, Mješovita srednja E. Pozderović	*20*	177.9 ±6.8	164.8-188.0	18.2 ±0.3	17.7-18.7

Abbreviations: SSŠ = Srednja strukovna škola (high vocational school).

Table S1b. Averages of boys in the measured schools (Republika Srpska).

**Region**<br>TOWN, school	*n*	Height (cm) Average (SD)	Height (cm) Range	Age (years) Average (SD)	Age (years) Range
**Region Prijedor**	***192***	**180.9 ±7.2**		**18.6 ±0.5**	
PRIJEDOR, Gimnazija Sveti Sava	*31*	183.2 ±7.4	171.2-199.4	18.6 ±0.4	17.5-19.3
PRIJEDOR, Elektrotehnička	*58*	181.7 ±7.6	164.9-200.3	18.5 ±0.5	17.6-19.5
PRIJEDOR, Medicinsko-tehnološka i gradev.	*33*	180.2 ±5.8	169.0-194.7	18.6 ±0.4	18.0-19.8
PRIJEDOR, Mašinska	*70*	179.5 ±7.0	166.8-201.0	18.5 ±0.5	17.6-19.6
**Region Banja Luka**	***170***	**180.6 ±6.6**		**18.4 ±0.5**	
BANJA LUKA, Gimnazija	*47*	181.7 ±6.8	170.9-200.4	18.7 ±0.4	18.1-20.2
BANJA LUKA, Ugost.-trgov.-turistička	*56*	180.3 ±7.0	165.3-198.0	18.2 ±0.5	17.3-19.2
BANJA LUKA, Poljoprivredna	*28*	180.1 ±5.9	170.8-194.3	17.9 ±0.3	17.4-18.3
BANJA LUKA, Elektrotehnička „N. Tesla“	*19*	180.0 ±6.4	167.0-193.5	17.8 ±0.4	18.2-19.4
BANJA LUKA, Tehnološka	*20*	179.8 ±5.8	167.3-194.0	18.9 ±0.6	18.1-20.3
**Region Mrkonjič Grad**	***80***	**180.3 ±6.2**		**18.3 ±0.6**	
MRKONJIČ GRAD, Gimnazija	*39*	180.6 ±7.4	167.2-197.2	18.1 ±0.6	17.0-19.6
MRKONJIČ GRAD, Mašinska	*41*	180.0 ±4.7	170.9-189.0	18.5 ±0.5	17.6-19.6
**Region Doboj**	***167***	**179.8 ±6.5**		**18.1 ±0.4**	
DOBOJ, Medicinska	*28*	180.4 ±6.7	166.0-199.6	18.1 ±0.5	17.1-19.5
DOBOJ, Saobracajna i elektro-škola	*55*	180.3 ±6.3	166.3-197.3	18.2 ±0.3	17.2-18.8
DOBOJ, Upravna & ugostiteljska	*32*	179.5 ±6.8	165.1-191.4	18.1 ±0.4	17.0-18.8
DOBOJ, Ekonomska & trgovinska	*16*	179.5 ±4.7	170.6-186.9	17.9 ±0.5	17.1-18.7
DOBOJ, Tehnička	*36*	178.8 ±6.8	164.5-195.3	18.1 ±0.5	17.0-19.5
**Region Bijeljina-Zvornik**	***233***	**181.2 ±6.8**		**18.0 ±0.5**	
BIJELJINA, Gimnazija Filip Višnjić	*32*	183.1 ±5.5	170.9-194.4	17.4 ±0.3	17.0-18.5
BIJELJINA, Tehnička Mihaljo Pupin	*67*	181.6 ±7.8	164.2-200.0	18.2 ±0.3	17.7-18.8
ZVORNIK, Gimnazija & stručna	*12*	181.1 ±5.7	168.6-190.3	17.3 ±0.2	17.0-17.6
BIJELJINA, Ekonomska	*34*	180.8 ±5.8	163.9-193.0	18.3 ±0.3	17.6-19.3
ZVORNIK, Ekonomska	*12*	180.8 ±6.4	164.3-189.6	17.8 ±0.5	17.1-18.5
UGLJEVIK, Sr. škola 'M. Petrović Alas'	*29*	180.2 ±5.6	166.2-192.8	18.3 ±0.3	17.7-18.9
ZVORNIK, Tehnički školski centar	*47*	180.1 ±7.1	165.6-197.8	18.1 ±0.4	17.3-18.8
**Region Istočno Sarajevo**	***60***	**184.0 ±5.5**		**18.1 ±0.4**	
ISTOČNO SARAJEVO, Gimnazija & stručna	*21*	185.9 ±4.9	176.8-198.0	18.1 ±0.3	17.5-18.6
ISTOČNO SARAJEVO, Sr. škola 28. juni	*39*	182.7 ±5.5	171.4-193.0	18.1 ±0.4	17.3-19.6
**Region Romanija-Foča**	***91***	**181.1 ±6.4**		**18.3 ±0.3**	
FOČA, SŠC	*11*	183.1 ±8.4	172.6-196.3	18.2 ±0.2	17.9-18.6
PALE, SSČ	*23*	181.4 ±6.5	169.6-195.4	18.4 ±0.4	17.9-19.5
ROGATICA, Srednja škola	*57*	180.5 ±5.9	166.8-189.9	18.3 ±0.3	17.8-19.5
**Region Trebinje**	***193***	**184.6 ±7.6**		**18.5 ±0.5**	
TREBINJE, Tehnička	*21*	187.0 ±7.3	174.7-200.0	18.7 ±0.4	18.0-19.2
TREBINJE, Gimnazija Jovan Dučić	*61*	185.7 ±7.3	175.4-206.6	18.3 ±0.5	17.4-19.3
NEVESINJE, SŠC Aleksa Šantić	*71*	184.5 ±7.1	164.4-201.6	18.6 ±0.4	18.1-20.4
TREBINJE, Centar srednjih škola	*40*	182.0 ±8.4	163.1-204.0	18.2 ±0.5	17.5-19.2

Abrreviations: SŠC = Srednjoškolski centar (high school center).

Table S2. Averages of girls in the measured schools.

TOWN, school	*n*	Average age (SD)	Height (cm)		Average height of boys from the same school	Difference in height between sexes
			Average (SD)	Range		
TOMISLAVGRAD, Gimnazija	*11*	17.9 ±0.2	171.0 ±5.6	158.8-180.5	184.3	13.3
POSUŠJE, Strukovna	*19*	-	169.4 ±4.5	161.8-180.7	183.9	14.5
GLAMOČ, Sr. škola Tin Ujević	*10*	17.7 ±0.3	169.3 ±5.3	161.9-178.7	178.8	9.5
DRVAR, Srednja škola	*11*	18.2 ±0.4	169.2 ±6.1	154.1-175.4	184.1	14.9
MOSTAR, Druga gimnazija	*18*	18.0 ±0.3	168.3 ±7.6	158.6-189.8	181.9	13.6
**TOTAL**	*69*	18.0 ±0.4	169.4 ±6.0	154.1-189.8		

Table S3a. Regional averages of male height (based on the self-reported place of residence) and population size in each region, relative to the total population of BiH (excluding Canton Posavina and the Brčko district).

Region	Average height (cm)	Population	% total
Region Trebinje	184.5	72,767	2.0
Canton Western Herzegovina	184.0	97,893	2.7
Region Istočno Sarajevo	184.0	30,077	0.8
Canton 10/Livno	183.7	90,727	2.5
Canton Herzegovina-Neretva	182.8	236,278	6.5
Canton Sarajevo	181.7	438,443	12.0
Canton Central Bosnia	181.7	273,149	7.5
Canton Zenica-Doboj	181.4	385,067	10.5
Region Bijeljina-Zvornik	181.2	286,880	7.9
Region Romanija-Foča	181.0	101,835	2.8
Region Prijedor	180.9	160,453	4.4
Canton Goražde	180.6	25,336	0.7
Region Banja Luka	180.5	399,469	10.9
Region Mrkonjić Grad	180.3	37,610	1.0
Canton Una-Sana	180.0	299,343	8.2
Canton Tuzla	180.0	477,278	13.1
Region Doboj	179.7	237,898	6.5
**BiH total**	**181.7**	**3,650,503**	**100.0**
Canton Posavina	-	48,089	-
Brčko district	-	93,028	-

Note: The data for „Region Istočno Sarajevo" were computed as an average of 4 municipalities (Istočna Ilidža, Istočni Stari Grad, Istočno Novo Sarajevo and Trnovo). Population size in each region was based on the preliminary results of the 2013 Census (http://www.bhas.ba/obavjestenja/Saopcenje_Preliminarni_rezultati_Bos.pdf).

Table S3b. Averages of male height corrected for population size in 17 regions.

	Average height (cm)	Population	% total	Average height corrected for population size
Bosnia total	180.9	3,152,838	86.4	180.8
Herzegovina total	183.6	497,665	13.6	183.4
Federation total	181.9	2,323,514	63.6	181.4
Republika Srpska total	181.4	1,326,989	36.4	180.9
BiH TOTAL	181.7	3,650,503	100.0	181.2

Table S4. Correlations between average male height and socioeconomic variables (for the year 2013) in 17 regions of BiH.

Variable 1	Variable 2	BiH (17 regions)	Bosnia (13 regions)	Herzegovina (4 regions)
Height	Net wage	0.43 (p=0.08)	0.34 (p=0.25)	-0.58 (p=0.42)
Height	Unemployment	0.28 (p=0.28)	0.22 (p=0.46)	0.42 (p=0.58)
Height	Agricultural activities (total)	-0.24 (p=0.35)	-0.48 (p=0.10)	0.94 (p=0.06)
Height	Agricultural activities (market-oriented)	0.11 (p=0.69)	-0.36 (p=0.23)	0.61 (p=0.39)
Net wage	Unemployment	0.07 (p=0.80)	-0.04 (p=0.91)	0.42 (p=0.58)
Net wage	Agricultural activities (total)	**-0.86 (p<0.001)**	**-0.93 (p<0.001)**	-0.68 (p=0.32)
Net wage	Agricultural activities (market-oriented)	-0.24 (p=0.35)	-0.49 (p=0.09)	0.30 (p=0.71)
Unemployment	Agricultural activities (total)	0.06 (p=0.83)	0.00 (p=0.99)	0.38 (p=0.62)
Unemployment	Agricultural activities (market-oriented)	0.23 (p=0.37)	-0.02 (p=0.94)	0.90 (p=0.10)
Agric. activities (total)	Agricultural activities (market-oriented)	**0.58 (p=0.015)**	**0.66 (p=0.014)**	0.43 (p=0.57)

Note: Significant correlations are in bold.

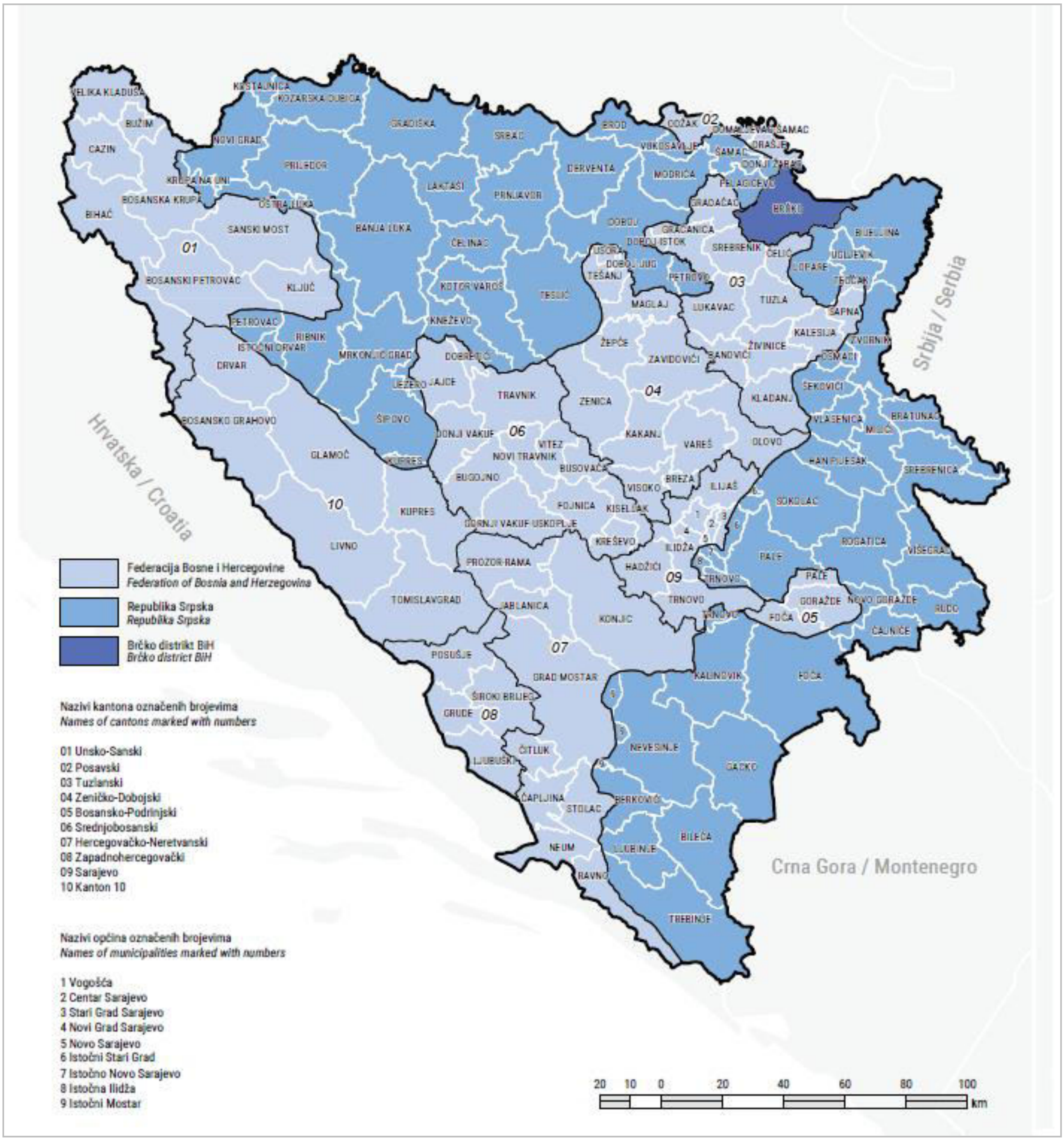

Figure S1. Administrative map of Bosnia and Herzegovina.

Source: Census of population, households and dwellings in Bosnia and Herzegovina, 2013. Final results. http://www.popis2013.ba/popis2013/doc/Popis2013prvoIzdanje.pdf

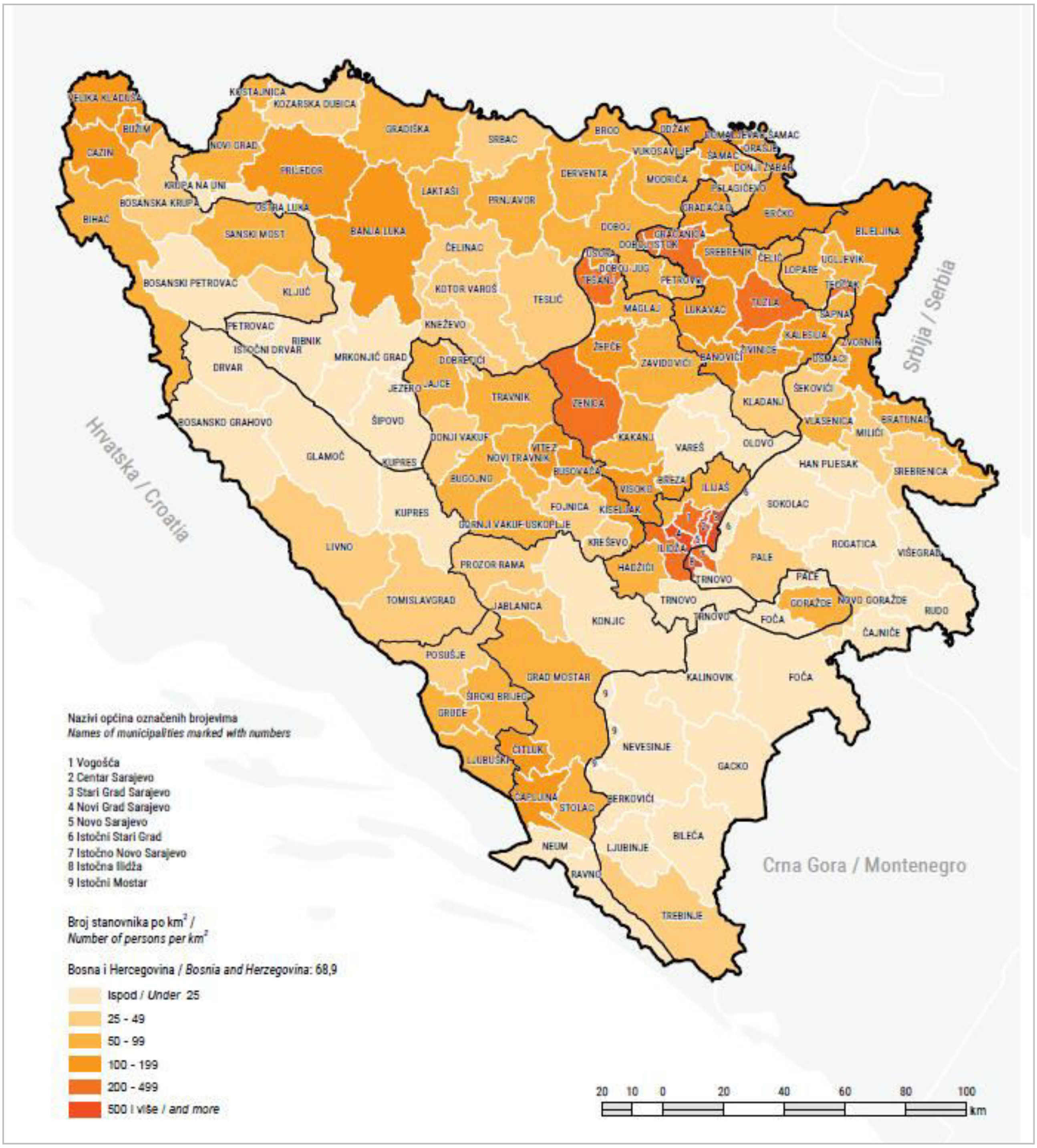

Figure S2. Population density in Bosnia and Herzegovina (people/km²).

Source: Census of population, households and dwellings in Bosnia and Herzegovina, 2013. Final results. http://www.popis2013.ba/popis2013/doc/Popis2013prvoIzdanje.pdf

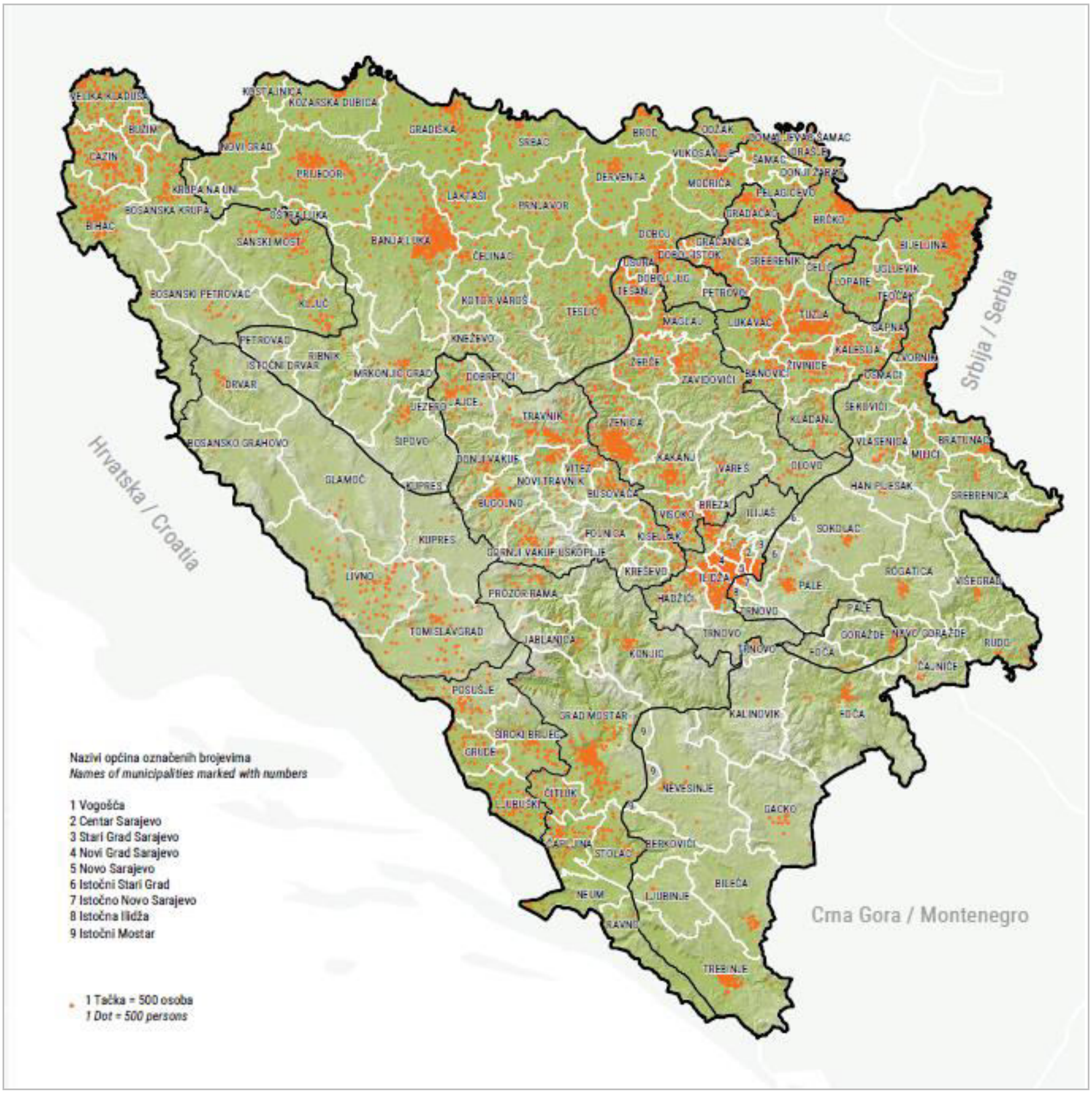

Figure S3. Density of settlements in Bosnia and Herzegovina.

Source: Census of population, households and dwellings in Bosnia and Herzegovina, 2013. Final results.
http://www.popis2013.ba/popis2013/doc/Popis2013prvoIzdanje.pdf

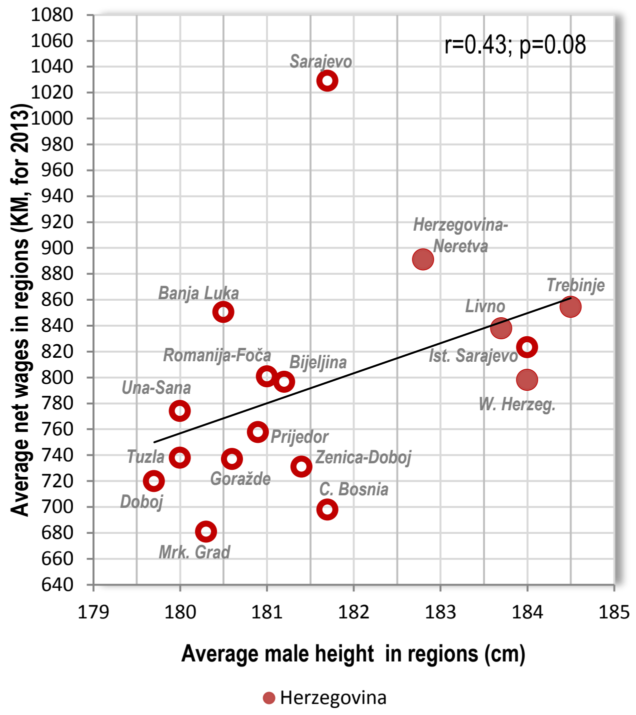

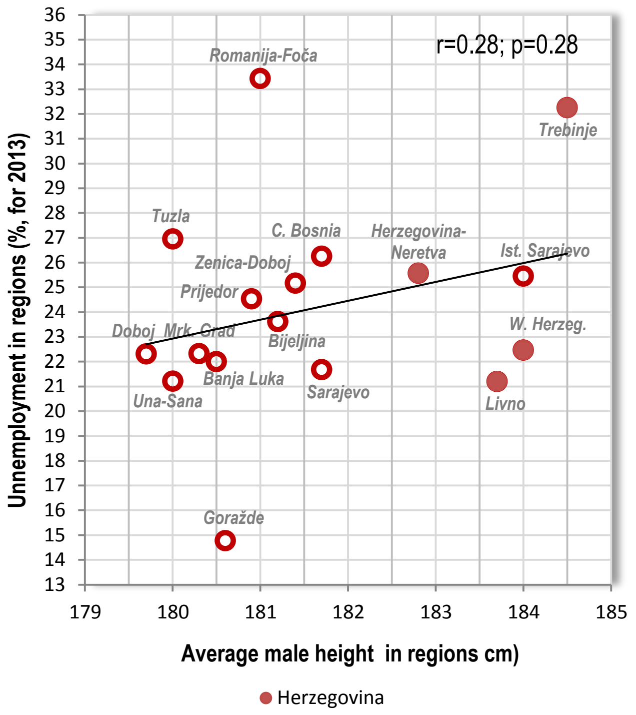

Figure S4b. Relationship between average male height and average net wages (for 2013, in KM/convertible marks) in 17 regions of BiH.

Figure S4b. Relationship between average male height and unemployment (for 2013) in 17 regions of BiH.

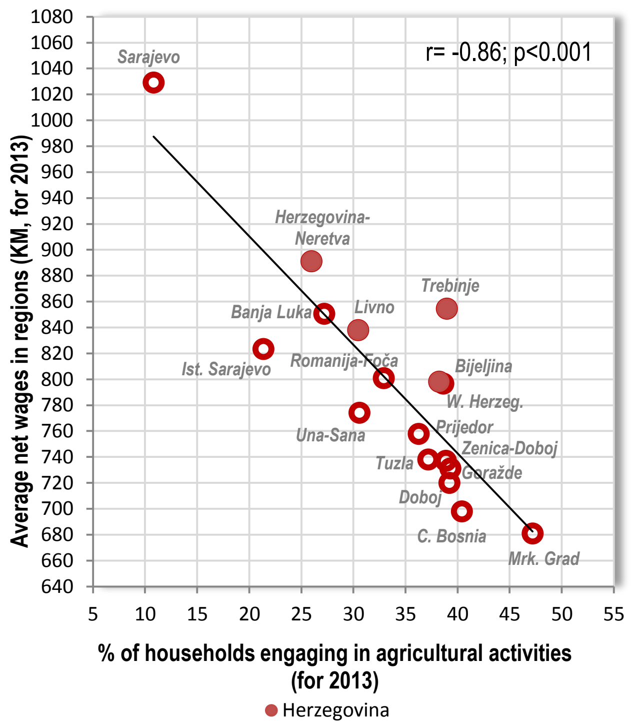

Figure S4c. Relationship between % of households engaging in agricultural activities (total) and average net wages (for 2013, in KM/convertible marks) in 17 regions of BiH.

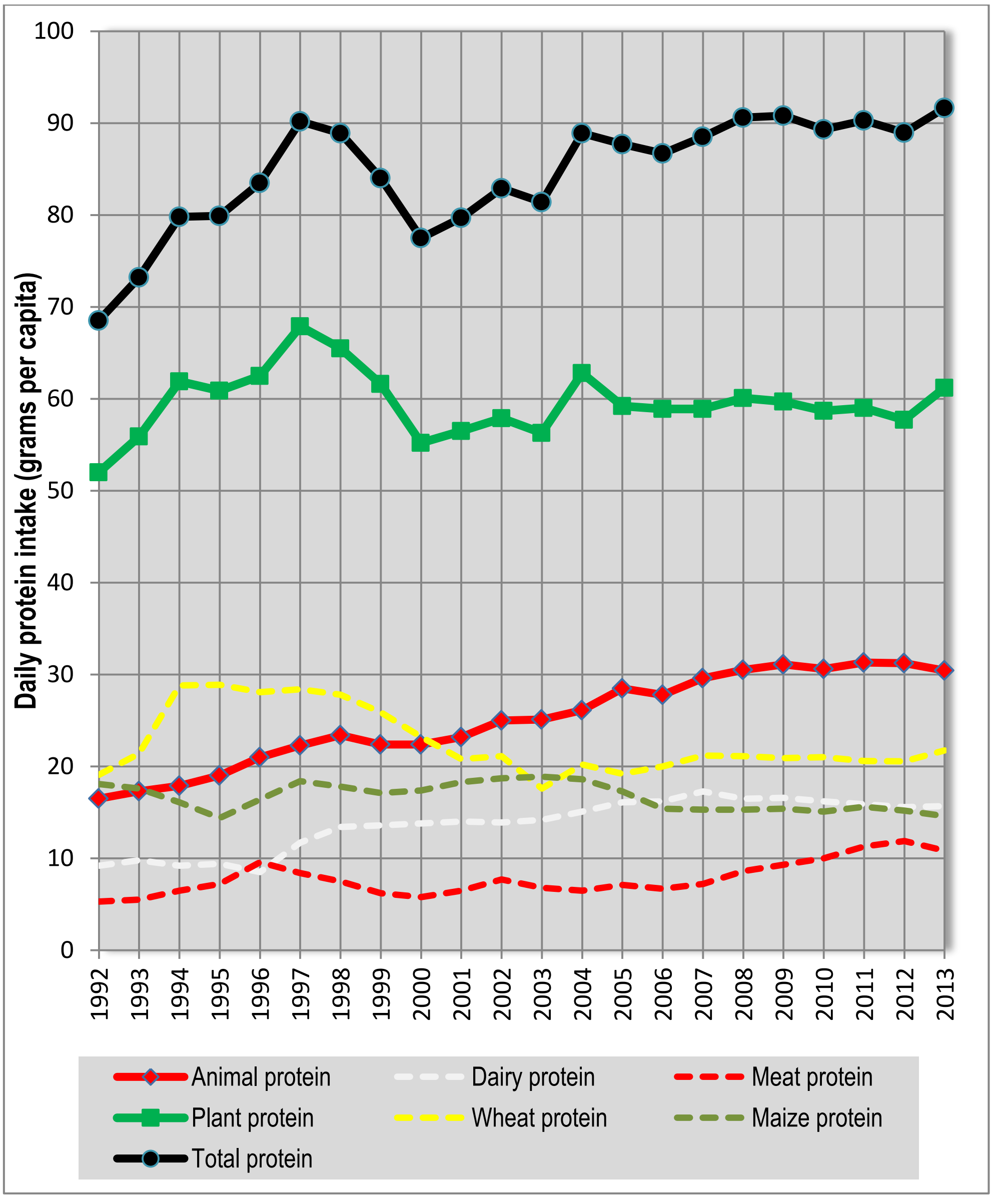

Figure S5. Average daily protein intake from major protein sources in BiH between 1992-2013.

Source: FAOSTAT, http://faostat3.fao.org/download/FB/CC/E.

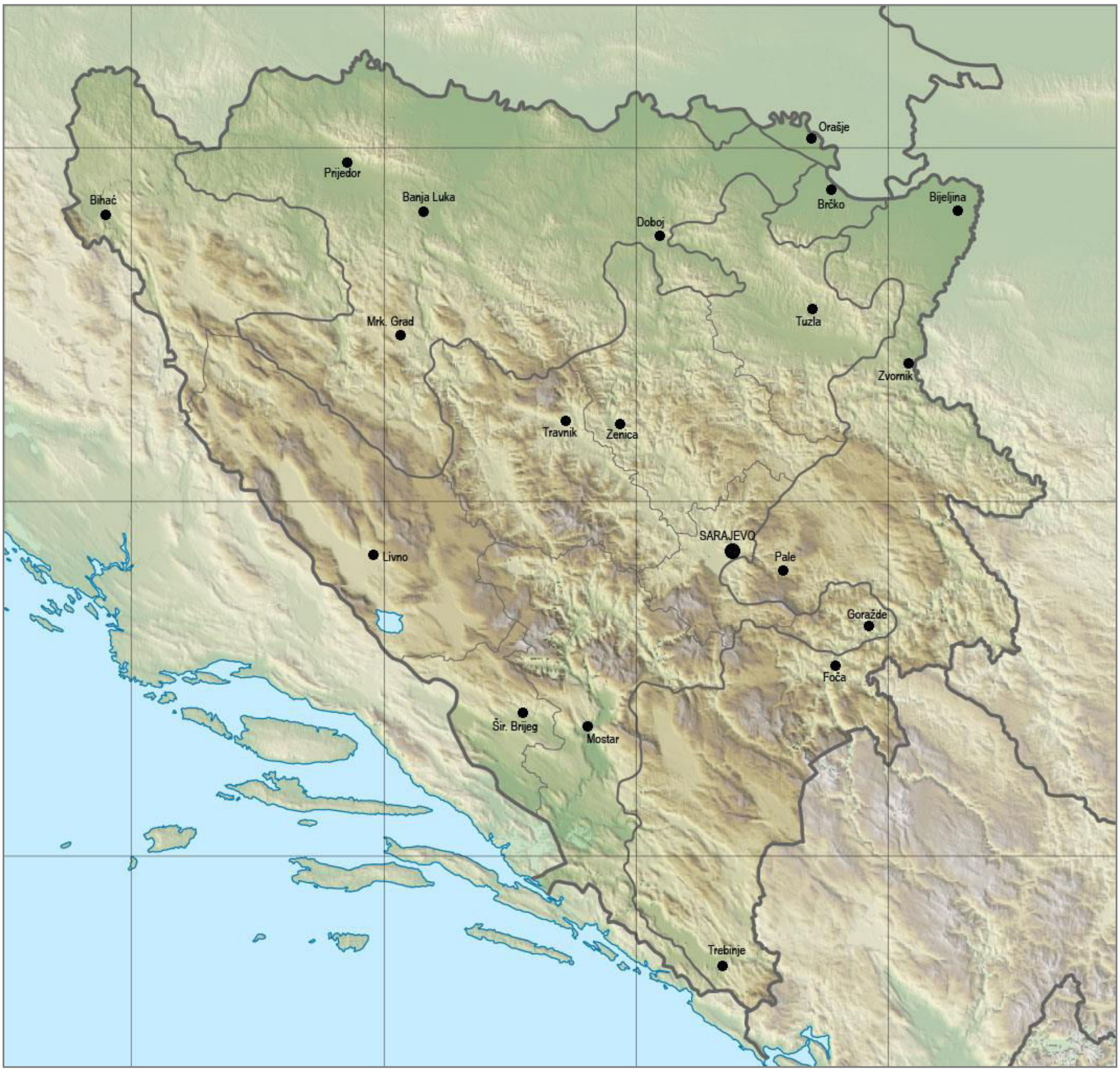

Figure S6. Relief map of Bosnia and Herzegovina.
Source: https://commons.wikimedia.org/wiki/File:Bosnia_and_Herzegovina_relief_location_map.png
